# Portraying the Expression Landscapes of B-Cell Lymphoma-Intuitive Detection of Outlier Samples and of Molecular Subtypes

**DOI:** 10.3390/biology2041411

**Published:** 2013-12-02

**Authors:** Lydia Hopp, Kathrin Lembcke, Hans Binder, Henry Wirth

**Affiliations:** 1 Interdisciplinary Centre for Bioinformatics, Universität Leipzig, Härtelstr. 16–18, Leipzig 04107, Germany; E-Mails: hopp@izbi.uni-leipzig.de (L.H.); lembcke@izbi.uni-leipzig.de (K.L.); binder@izbi.uni-leipzig.de (H.B.); 2 LIFE, Leipzig Research Center for Civilization Diseases, Universität Leipzig, Philipp-Rosenthal-Straße 27, Leipzig 04103, Germany

**Keywords:** co-regulated genes, molecular function, network analysis, machine learning, classifying cancer

## Abstract

We present an analytic framework based on Self-Organizing Map (SOM) machine learning to study large scale patient data sets. The potency of the approach is demonstrated in a case study using gene expression data of more than 200 mature aggressive B-cell lymphoma patients. The method portrays each sample with individual resolution, characterizes the subtypes, disentangles the expression patterns into distinct modules, extracts their functional context using enrichment techniques and enables investigation of the similarity relations between the samples. The method also allows to detect and to correct outliers caused by contaminations. Based on our analysis, we propose a refined classification of B-cell Lymphoma into four molecular subtypes which are characterized by differential functional and clinical characteristics.

## 1. Introduction

Cancer is a complex disease caused by the deregulation of gene activity. Carcinogenesis and -progression is accompanied by dysfunctions on multiple layers of the cellular machinery. They are affected by a large number of different genetic and epigenetic factors. In recent years, large-scale studies such as The Cancer Genome Atlas (TCGA) [1,2], The Cancer Cell Line Encyclopedia [3] or the International Cancer Genome Consortium (ICGC) [4] were undertaken aimed at characterizing cancer on the molecular and cellular level. These studies allowed to discover the heterogeneity of the underlying regulatory mechanisms and to assign them to molecular cancer subtypes. 

On the one hand, high-throughput technologies such as whole genome transcriptional profiling presently revolutionize molecular biology and provide an incredible amount of data. On the other hand, these techniques pose elementary methodological challenges simply by the huge and ever increasing amount of data produced [5,6,7,8]: researchers need adequate tools to extract the information content of the data in an effective and intelligent way. This includes algorithmic tasks such as data compression and filtering, feature selection, linkage with the functional context, and proper visualization. 

Especially, the latter task is very important because an intuitive visualization of massive data clearly promotes quality control, the discovery of their intrinsic structure, functional data mining and finally the generation of hypotheses. We aim at adapting a holistic ‘systems’ view on the gene activation patterns as seen by expression studies rather than to consider single genes or single pathways. This view requires methods which support an integrative and reductionist approach to disentangle the complex gene-phenotype interactions related to cancer genesis and progression. 

With this motivation we apply Self-Organizing Maps (SOM), a machine-learning clustering approach [9], to a large-scale patient expression data set of mature aggressive B-cell lymphomas published previously [10]. Our approach simultaneously searches for features which are differentially expressed and correlated in their profiles in the set of samples studied [11]. We include functional information about such co-expressed genes to extract distinct functional modules inherent in the data and attribute them to particular types of cellular and biological processes such as inflammation, cell division, *etc*. [12]. This modular view facilitates the understanding of the gene expression patterns characterizing different cancer subtypes on the molecular level. Importantly, SOMs preserve the information richness of the original data allowing the detailed study of the samples after SOM clustering [11]. 

A central role in our analysis is played by the so-called expression ‘portraits’, which serve as intuitive and easy-to-interpret fingerprints of the transcriptional activity of the samples. Their analysis provides a holistic view on the expression patterns activated in a particular sample. Importantly, they also allow identification and interpretation of outlier samples and, thus, improve data quality. 

Our application of SOM machine learning to lymphoma expression data aims at characterizing the heterogeneity of the genome wide expression landscapes and at describing the molecular cancer subtypes. In particular, we will demonstrate the capabilities of our strategy to intuitively visualize the individual samples as well as the subtypes in terms of individual and group-averaged portraits, respectively. We show how to extract functional information from the data and appropriately incorporate it into the analysis strategy. Further, we describe how to detect and to correct outlier samples using their portraits. Finally, we propose a more detailed molecular subtype classification of the lymphoma samples.

## 2. Data and Methods

### 2.1. Expression Data and Preprocessing

Microarray data of lymphoma are available under GEO accession number GSE4475 (data from 221 Affymetrix HG-U133A arrays). This study used biopsy specimens of mature aggressive B-cell lymphoma in which at least 70 percent of all cells were tumor cells. The classification of lymphoma samples into different subtypes is used as provided by Hummel *et al*. [10]: Of all 221 lymphomas, 44 are assigned to the mBL (molecular Burkitt’s lymphoma) signature and 129 to non-mBL signature. 48 cases form an intermediate group, representing the transition zone between the mBL and non-mBL groups.

For a convention we use the data as numerical matrix of dimension *N* × *M* where *N* is the number of genes measured per sample and *M* is the number of samples in the study. Throughout this paper a row of this matrix will be termed ‘expression profile’ of the respective gene. The columns on the other hand will be termed ‘expression states’ referring to one sample studied.

Raw probe intensity values of Affymetrix arrays were calibrated and summarized into one expression value per probe set using the hook method [13,14]. To ensure comparability, we applied quantile-normalization to the samples [15]. It transfers the expression states of all samples into one common distribution. Then, the expression values of each gene were transformed into log10-scale and centered with respect to the mean expression value of the particular gene averaged over all samples in the study [11]. This translates the expression data into fold change units and will be addressed as log*FC =* Δ*e_i,m_*, the relative log-expression of gene *i* in sample *m*. Hence, a Δ*e_i,m_* of zero means that the gene is expressed according to its mean expression value. Positive and negative values refer to over- and under-expression in the series of samples, respectively.

### 2.2. SOM Training

The preprocessed expression values Δ*e_i,m_* are used to train a Self-Organizing Map (SOM). It translates the high-dimensional *N* × *M* expression data matrix into a *K* × *M* metadata matrix (*K*: number of so-called metagenes, in literature also referred as ‘nodes’, ‘units’, ‘prototypes’ or ‘cells’ of the SOM) of reduced dimensionality *K* << *N* (*N* = 22,283 and *K* = 2,500). The corresponding relative log-expression values of the metagenes will be termed Δ*e_k,m_^meta^*. The metagene expression profiles (in literature also named ‘weight vectors’ or ‘prototype vectors’) are adapted in the iterative machine learning process to optimally cover the data space once the training is completed ([9], see [16] for detailed illustration). Therefore, the metagene profiles are slightly altered in each iteration such that they resemble the input gene profiles more closely. Each metagene serves as a representative prototype of a cluster of real genes with similar expression profiles. The metagenes’ expression profile in turn approximately resembles the average profile over the associated real genes. Note that during training, the association of genes to the metagenes is not fixed and alters in a self-organizing process with the effect that the degree of similarity between metagenes decreases with increasing distance in the trained map.

Our SOM method was configured to enable the robust identification of spot modules inherent in the data (see below). Details were described previously [11,12,17]. In short: We have shown that the particular choice of the grid topology (e.g., rectangular or hexagonal) and of the map size (if chosen between *K* = 30 × 30 and *K* = 60 × 60 metagenes) is not crucial for downstream expression analysis. It provides almost identical results in terms of the expression patterns identified (see [11] in the supplementary material, and [16]). Variation of the SOM-size in reasonable limits can slightly alter the smoothness of the expression landscapes observed but not their basal properties required for further analysis [11,16]. Our choice of SOM size is further supported by an independent heuristic based on the two largest eigenvectors to estimate the map size [18]: The use of its implementation in ‘SOM toolbox 2.0’ returns an optimal SOM size of *K =* 42 × 28 metagenes.

In this application, we used a two-dimensional grid of size *K* = 50 × 50 metagenes and of rectangular topology, Gaussian neighborhood function [11,16], and the implementation of the algorithm in the R-package ‘som’ [19].

### 2.3. SOM Staining

Each sample’s meta-state is described by the *K* expression values in the columns of the metadata matrix. They are arranged according to the underlying metagene grid and visualized by an appropriate color gradient: dark red reflects strong over-expression; yellow and green tones indicate intermediate levels with low or no differential expression; and blue corresponds to under-expression. The color patterns emerge as smooth textures representing the fingerprint of transcriptional activity of each sample. Please note that the assignment of the genes to metagene clusters and therefore also their position in the SOM is identical in all sample portraits. Hence, the coloring at a certain position in the map refers to the same genes in all individual portraits allowing the direct comparison of their expression levels between the maps.

Subtype-specific mean portraits are calculated and visualized as the mean value of each metagene averaged over all sample portraits belonging to one subtype. They reflect subtype specific expression patterns while leveling out the heterogeneity of the individual expression states and outliers.

### 2.4. Detection of Expression Modules: Spot Selection

The SOM algorithm arranges similar metagene profiles in neighbored tiles of the map whereas more different ones are located more distantly. Adjacent metagenes thus tend to be colored similarly and the obtained mosaic portraits show typically smooth patterns with red and blue spot-like regions referring to clusters of over- and under-expressed metagenes, respectively. Metagenes located in the same spot are concertedly expressed across the samples studied. Consequently, distinct and well-separated spots in one sample collect genes of different expression profiles although concertedly over-expressed (or under-expressed) in this particular sample. Each spot can consequently be interpreted as a disjunct expression module of a group of metagenes (and of associated single genes) showing a unique expression profile in the data set studied.

We define over-/under-expression spots by applying a simple 98th/2nd-percentile criterion as described and verified in [11,12,20,21,22,23]. It selects the respective fraction of metagenes showing largest/smallest expression in each sample. The percentile criterion chosen allows selection of a sufficient number of candidate genes per spot on one hand and a sufficient number of relevant spots on the other hand. Moderate modifications of the percentile criterion used are uncritical with respect to the final results obtained. All spots detected in the individual portraits are transferred into one master map to visualize the global spot patterns of the series evaluated. This provides a simple and intuitive approach for the detection of expression modules inherent in the data. Note that this detection of spot modules provides gene clusters in an unsupervised fashion without necessity for prior definition of prototypes or cluster numbers.

We further implemented and verified complementary methods of spot selection using different metrics and algorithms such as k-Means and hierarchical clustering based on Euclidean distance between the metagene profiles, and seed-clustering based on their pairwise correlation coefficient [11,12,16]. The basal functional impact of the modules obtained is virtually independent of the particular method of spot selection used although the spots can differ considerably in the number of genes and the area of the map included. We here apply the over-/under-expression spot selection method because it selects lists of strongly differentially expressed genes. Such lists are of particular interest not only in our analysis but also in numerous gene expression studies aiming to detect marker genes.

### 2.5. Enrichment Analysis

Co-expressed genes of each spot module can be assumed to be functionally related according to the ‘guilt-by-association’ principle [24]. Functional analysis aims at identifying the functional context of these expression modules. 

We use different approaches to estimate the enrichment of groups of predefined genes (so-called gene sets) in gene lists obtained independently, for example from SOM-spot analysis (see [25] for a critical review). Enriched gene sets indicate an association between their context and the system studied. A large and diverse collection of such gene sets can be derived from the Gene Ontology (GO) annotation database [26] using the ‘biomaRt’ interface [27]. In particular, a total of 5,154 gene sets are included in our analysis according to the following categories: (i) 1748 GO gene sets subdivided into GO-terms ‘biological process’ (1,102 sets), ‘molecular function’ (387 sets) and ‘cellular component’ (259 sets); (ii) pathways referring to Biocarta (217 sets), KEGG (186 sets) and Reactome (430 sets) databases; (iii) curated gene sets taken from the literature on chemical and genetic perturbations (‘literature sets’, 2,439 sets); (iv) tissue specific gene sets (25 sets) derived previously from a gene expression study on healthy human tissues [12]; and (v) ‘special’ gene sets taken from the literature on various cancer types and subtypes (109 sets).

Under the term ‘enrichment analysis’ we here subsume ‘overrepresentation’ and combined ‘overrepresentation’/‘overexpression’ analyses (see references [12,28] for a detailed discussion). In our approach, overrepresentation estimates the probability of finding more members of a given gene set in a particular spot cluster compared with their random appearance, independent of their expression values. Right-tail modified Fisher exact test and the hypergeometric distribution then provide a *p*-value for each predefined gene set in each spot. This *p-*value reflects the overlap between the genes in a spot cluster and the gene set given a certain total number of genes studied [12,29,30]. We considered overrepresented sets with *p* < 10^−5^ which ensures reasonable adjustment for false positives in multiple testing. In particular, this criterion applies Bonferroni adjustment *p* < *α/n*, where *α* denotes the desired significance level and *n* is the number of single tests. With *α* = 0.05 and *n* ≈ 5000 (number of gene sets tested) one obtains *p* < 10^−5^. Note that Bonferroni adjustment represents a conservative approach minimizing the family wise error rate (see e.g., [31]). However, it applies to statistically independent tests, a requirement which is not given for many gene sets used because they contain a high percentage of overlapping genes. The criterion applied therefore provides a conservative lower limit of acceptable gene sets.

As a second approach, ‘over-expression’ defines the deviation between the mean expression value averaged over the set-members compared with the mean expression value of all genes measured in a sample. The so-called gene set Z-score (GSZ) combines both options of gene set overrepresentation and overexpression approaches [12,32]. In particular, the GSZ-score for the list of all genes studied is given by

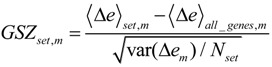
(1)
where ˂Δ*e*˃_*set,m*_ is the mean expression of the gene set members in sample *m*, ˂Δ*e*˃_*all genes,m*_ denotes the mean expression of all genes and the denominator defines the respective standard error (for a detailed description see [12]). We use the GSZ-score to profile enrichment of a selected gene set across all samples and cancer-subtypes studied.

In addition to overrepresentation in spots and the GSZ-profiles, we generate gene set population maps. They visualize the distribution of the genes of a selected set in the SOM grid by appropriate color coding of the number of set members assigned to each metagene. It ranges from white (no gene) to maroon (maximum number of genes per tile observed for the particular gene set). Recall that each gene refers to one and the same metagene in all samples and thus occupies a fixed position in all SOM portraits allowing comparison between gene positions and spot positions in reference to specific functional modules.

### 2.6. Sample Similarity Analysis

Sample similarity analysis aims at evaluating mutual relations between the samples studied. There are various established approaches, for example to extract a hierarchy of similarities, to estimate mutual distances between the expression states or to assess the main sources of variance in the data. 

Here, we use three different metrics, namely statistical dependence, Euclidean distance and Pearson’s correlation, which are applied to the metadata instead of to the original ‘single gene data’. Similarity analysis consequently compares the expression meta-states as characterised by the SOM portraits. The usage of metadata as the basal data has the advantage of improving the representativeness and resolution of the results as shown previously [11,20,33].

Independent component analysis (ICA) [34] is applied to the SOM-metadata using the R-package ‘fastICA’ [35]. It distributes the samples in the space spanned by the components of minimal mutual statistical dependence. These components point along the directions of maximum information content in the data which is estimated by their deviation from a (non-informative) normal distribution [34]. 

As a second option, we apply the neighbor-joining algorithm (R-package ‘ape’ [36]) to visualize similarity relations based on the Euclidean distances between the samples as similarity trees [37]. The distances between pairs of samples in the tree are in scale. It allows to identify ‘bush-like’ clusters and to estimate the degree of mutual dissimilarity between them.

Pearson product-moment correlation is the third metric we use. It calculates correlation coefficients between the metagene states for all pairwise combinations of samples. The resulting quadratic correlation matrix is visualized by coloring the correlation values in the pairwise correlation map (PCM) using a color gradient ranging from red for positive correlation to blue for negative correlation. The correlation network (CN) additionally translates the correlation matrix into a graph structure. This undirected graph is constructed by connecting the nodes (*i.e.*, the samples), whose pairwise correlation coefficient exceeds a given threshold. Here, we chose *r_threshold_* = 0.5, which ensures a relatively sparse but still fully connected graph structure (see description in Supplementary File 1). It provides a network-like overview about the correlation structure of the expression landscapes of the samples. It is capable to intuitively display multivariate relations in contrast to univariate dendrograms. The lengths of the edges in the CN approximately scale inversely with the respective degree of correlation.

### 2.7. Correction of Biased Data

Systematic deviations give rise to biased data. They can be caused, for example, by individual specifics of the expression characteristics of the patients not related to the disease, by the inaccurate biopsy of the tumor cells leading to contaminations of the samples with healthy tissue or by systematic variations in the sample preparation process. Inspection of the individual SOM portraits combined with similarity and gene set enrichment analyses provide a framework of hand-in-hand options to detect and to correct strongly biased samples: Firstly, outlier spots can be detected in the portrait gallery and subsequently analyzed for their functional context in terms of overrepresented gene sets. Secondly, outlier samples can be identified in the correlation network similarity plot and then further evaluated by functional spot analysis. Gene sets found to be associated with outlier spots and/or samples are then simply excluded from further analyses leading to corrected expression portraits. This procedure can be repeated for different putative sources of systematic errors.

### 2.8. Molecular Subtypes Derived from Prototype-Guided k-Means and from Consensus Clustering

Identification of distinct molecular phenotypes is a common and important question in cancer research. A previous study of lymphoma data classifies the samples into the main subtypes molecular Burkitt’s Lymphoma (mBL), non-mBL and an intermediate group [10]. Both, inspection of SOM portraits and their similarity analyses suggests the further refinement of this classification into four subtypes, namely mBL*, non-mBL*, *intermediate-A* and *intermediate-B* (see below).

We applied a modified ‘prototype-guided’ k-Means clustering of the metadata to segregate the samples into these four subtypes. k-Means is an iterative algorithm which iteratively assigns the samples to so-called cluster prototypes showing the minimal mutual Euclidean distance and subsequently computes new prototypes as the centroids of the members of each cluster [38]. k-Means requires predefinition of a desired cluster number, while the initial prototypes are usually chosen randomly or initialized from the data [39]. 

The SOM portraits now constitute another option to initialize the prototypes: they can be established using selected expression patterns observed in the portraits such as the most prominent overexpression spots. Particularly, we define initial prototypic expression portraits showing a selected spot pattern for each subclass with values ‘max(*Δe_k,m_^meta^*)’ for metagenes within the spot and ‘0’ for metagenes outside. These prototypic spot patterns are then used to assign the samples to the respective clusters in the standard k-Means algorithm. Then, a bootstrapping approach is used to estimate the robustness of the assignment of samples to the subtypes. Therefore, k-Means clustering is repeatedly applied to a subset of samples chosen randomly from the complete set of samples. The mean metagene expression states of the subtypes are used as initial cluster prototypes. The fraction of proper assignments of samples in agreement with their actual class assignment then defines a robustness score of each sample: a bootstrap stability score of ‘1’ means that the respective sample is always found in the correct subtype, while a score of ‘0.5’ means that the sample is assigned properly in only 50% of the resampling repetitions.

In addition, we applied consensus clustering [40] to validate the results of our k-Means approach by an independent method. Consensus clustering aims at reaching a consensus on the number of classes in the data and at judging reliability of the class assignment of the samples. We applied the R-package ‘ConsensusClusterPlus’ [41] for portioning the samples into *k* classes using hierarchical clustering with *k* ranging from two to six. For each *k*, one obtains a consensus matrix, reflecting the fraction of common class memberships for all pairwise combinations of samples estimated in a series of resampling runs (details are given in [40]). It is visualized by means of a clustered heatmap collecting samples frequently found in one class into blue squares along the diagonal. The cumulative distribution function (CDF) aggregates the consensus values up to a certain fractional co-occurrence of sample pairs. The CDF thus reflects the ‘degree of heterogeneity’ of a consensus matrix using one curve such that clusterings with different *k* can be directly compared with the purpose to identify the optimal class number [40]. The incremental change between CDF curves with increasing *k* serves as a measure to judge whether increasing the class number leads to a marked increase of clusters’ stability or not. 

### 2.9. Additional Expression and Phenotypic Data

Gene expression data from germinal center B-cell line samples and tissue samples of tonsils were taken from reference [42]. After preprocessing as described above, these data were co-trained with the lymphoma data to evaluate the cell of origin characteristics of the lymphoma samples. Recently published patient phenotypic data were used to characterize the newly defined subtypes in the cohort studied [43]. These included data from immunohistochemical staining against CD10, BCL2, BCL6, MUM1, data from interphase fluorescence *in situ* hybridization (FISH) for IGH, MYC, BCL6 and BCL2 loci, overall survival, age and gender.

## 3. Results and Discussion

### 3.1. SOM Expression Portraits of Lymphoma Samples and Subtypes

SOM machine learning transforms the whole genome expression pattern of the ‘single’ genes into metagene expression data. Thereby, the number of single genes exceeds the number of metagenes by about one order of magnitude (*N* = 22,283 and *K* = 2,500). We visualize the expression meta-state of the samples as mosaic images, consisting of 50 × 50 tiles each representing one metagene. These metagenes serve as representatives of clusters of co-expressed single genes the number of which usually varies from metagene to metagene. The color gradient of the portraits was chosen to visualize over- and under-expression of the metagenes in each particular sample: red to green colors indicate over-expression with decreasing strength, while blue to green colors indicate under-expression. The colored texture of each mosaic thus individually characterizes the gene expression landscape in each sample.

**Figure 1:**
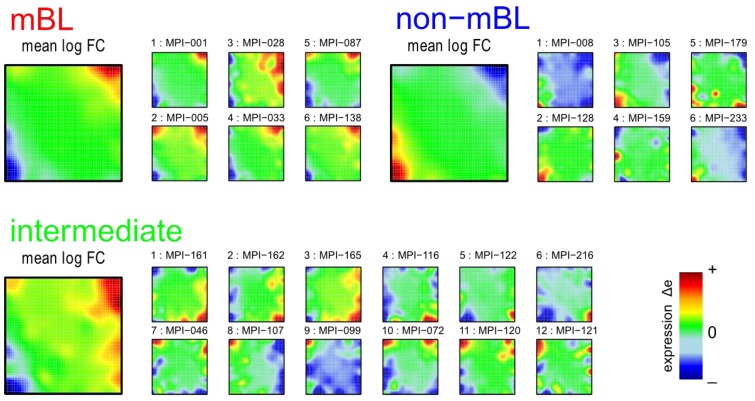
Self-organizing map (SOM) gallery of lymphoma subtypes with a resolution of 50 × 50 metagenes: The small mosaic images refer to selected individual tumor samples assigned to the mBL, non-mBL and intermediate subtypes. The larger images represent the respective mean subtype portraits (see methodical section). Dark red/blue colored metagenes refer to the 90th/10th-percentile of expression in each sample, respectively. The complete gallery of all sample portraits is available in Supplementary File 2.

Figure 1 shows the expression portraits of selected lymphoma samples arranged according to their previous classification into subtypes [10]. The individual portraits reveal a handful of clusters of co-expressed metagenes frequently observed. These so-called over- and under-expression spots selectively characterize the different lymphoma subtypes: samples of the mBL and non-mBL subtypes are mostly characterized by spots of overexpressed metagenes in top*-*right and bottom-left corners of the map, respectively. However, many additional spots can be observed in the portraits, indicating additional functional modules activated in the respective samples (see below). Samples of the intermediate subtype show more volatile patterns with over-expressed metagenes frequently tending to occupy the top*-*left and bottom-right corners of the SOM. The full gallery of the 221 SOM portraits is given in Supplementary File 2. Supporting maps characterizing the population of metagene clusters with single genes and the variance of the expression profiles of the metagenes are provided in Supplementary File 1.

We generate mean subtype portraits by averaging the expression values of each metagene over all the subtype members. This averaging cancels out the highly fluctuating, individual features and, thus, amplifies consistent subtype-specific features. In support of the observations from the individual portraits we found that the mBL and non-mBL subtypes are characterized by two spots in opposite corners of the map: one spot in the top*-*right corner is over-expressed and the other one in the bottom-left corner is under-expressed in mBL samples and *vice versa* in non-mBL samples, revealing the antagonistic character of their expression patterns. These subtype-specific spots collect highly populated, highly variable and well resolved metagenes (see Supplementary File 1).

In summary, SOM expression portraits reflect the individual expression landscapes of each sample in terms of characteristic color textures which enable visual perception of subtype-specific spot-like features representing clusters of differentially and co-expressed genes.

### 3.2. Characterizing the Expression Modules: Spot Analysis

Standard analysis tools usually evaluate the whole expression states of the individual samples to perform similarity or cluster analyses, or to generate lists of differentially expressed genes. Such global comparisons might overlook subtle effects due to individual properties of small groups of genes. These details are however projected into the color textures of the individual SOM portraits which change from sample to sample and can be assessed by means of feature selection (see [12] for a detailed review). The most prominent patterns are the over- and under-expression spots formed by neighboring metagenes of similar profiles which, in turn, represent clusters of correlated and thus potentially co-regulated genes strongly over- and/or under-expressed in a subset of samples.

**Figure 2:**
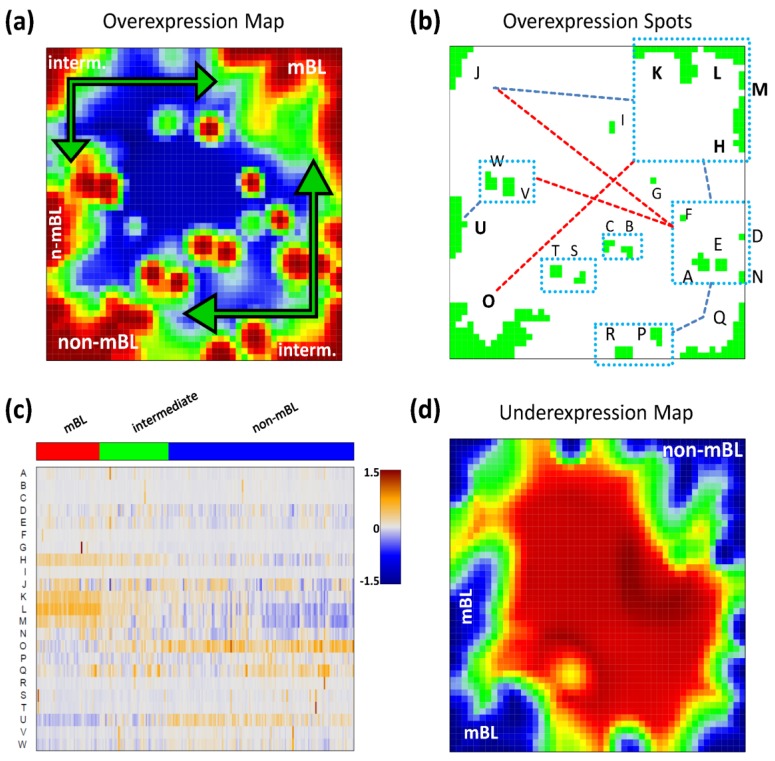
Spot module characteristics: (**a**) The over-expression summary map collects all over-expression spots observed in the individual portraits into one map. Subtypes frequently showing the respective spots are indicated. (**b**) The over-expression spot map defines the spots used for further analysis. Regions beyond the 98th-percentile threshold of metagene expression are selected. The spots are assigned by large capital letters. The blue rectangles include highly correlated spots (*r* > 0.7). The blue and red dashed lines connect correlated (0.4 < *r* < 0.7) and anti-correlated (*r* < −0.6) spots, respectively. (**c**) The overexpression heatmap shows the mean expression of the spots across all samples in the data set. The samples are sorted according to their subtype. (**d**) The under-expression summary map collects all under-expressed spots observed in the individual portraits. Note the antagonistic nature of mBL and non-mBL expression: spots over-expressed in mBL become under-expressed in non-mBL and *vice versa* (compare with panel a).

We analyze the spot patterns in order to identify specific properties of the lymphoma subtypes. Figure 2a shows the so-called over-expression summary map which collects all over-expression spots observed in the individual sample portraits into one master map (see also [11]). Each disjunctive region of this map exceeding the 98th-percentile threshold defines one global overexpression spot. It represents a distinct expression module inherent in the data. In total, we identified 23 over-expression spots labeled with capital letters ‘A’–‘W’ (Figure 2b).

Please note that our spot selection algorithm neglects the abundance of each spot in the individual portraits and identifies both rare (e.g., observed in only one sample) and frequent spot modules. The over-expression heatmap in Figure 2c visualizes the spot expression profiles, *i.e.*, the mean expression level of the metagenes in each of the spots across all samples. The colors range from blue representing the lowest mean expression values, to red representing the highest values. The samples are arranged according to their subtype classification. The heatmap provides an overview of the degree of subtype-specific expression in each of the spot modules. For example, spots ‘L’ and partly also spot ‘K’ are selectively over-expressed in samples of the mBL subtype, while spot ‘O’ is characteristic for the non-mBL subtype. Contrary, more ubiquitous spots as ‘N’, as well as rare spots as ‘A’ or ‘G’, lack of subtype-specific overexpression. Note that frequent spots are usually located in the peripheral part of the map (*i.e.*, in the corners and along the edges) whereas rare spots tend to accumulate in the central part.

We use the spot information and the mean subtype portraits to assign subtype labels to the most prominent and specific spot modules (Figure 2a): Spots ‘L’ and ‘K’ are ascribed to mBL while spot ‘O’ is prominent in non-mBL. Those three spot modules contain marker genes over-expressed in the respective subtypes as validated below. Spots ‘J’ and ‘Q’, also frequently observed in the sample portraits, are assigned to the intermediate subtype. Interestingly, they constitute two alternative intermediate states located in between the main-subtypes mBL and non-mBL. They are characterized either by spot ‘J’ or by spot ‘Q’ as indicated by the arrows in Figure 2a.

Please note that the training algorithm distributes the metagenes in such a way that strongly correlated profiles are located at adjacent positions in the map whereas metagenes with anti-correlated profiles tend to occupy more distant regions, e.g., in the opposite corners of the map. This rule also applies to the spots detected. In order to discover the covariance between the spot modules we calculated Pearson correlation coefficients for all pairs of spot profiles. It turned out that, as a rule of thumb, neighboring spots are strongly positively correlated and spots located in opposite corners of the map are often strongly anti-correlated. The results of this correlation analysis are visualized in Figure 2b. One sees that, for example, the mBL marker spots ‘K’ and ‘L’ are highly correlated and usually appear together in the sample portraits whereas the anti-correlated over-expression spots ‘K’ and ‘O’ will not be observed together in the same expression portrait. 

For this dataset, we also detected 11 global under-expression spots emerging as blue regions in the SOM portraits. The under-expression summary map is shown in Figure 2d. Position and size of most of the detected under-expression spots agree with those of the over-expression spots. Hence, overexpression of the respective metagenes in part of the samples changes into under-expression in other samples. For the analyses described in this paper, we therefore use only the over-expression spots detected without loss of essential information. Interestingly, virtually no blue under-expression spot was detected in the central area of the map indicating that the rare over-expression spots do not show this dualism. Below we will show that these spots potentially constitute clusters of outlier genes the expression of which is affected by bias effects.

In summary, the heterogeneous expression patterns observed in the individual portraits can be condensed to a few major expression modules represented by over- and under-expression spots. This way the relevant dimension of the data set is reduced by three orders of magnitude from about 20,000 single genes to approximately 12 spot modules.

### 3.3. Mining the Functional Context: Gene Set Enrichment Analysis

Each global overexpression spot module represents a cluster of potentially co-regulated genes. We applied gene set overrepresentation analysis to each spot-cluster taking into account a collection of more than 5,000 predefined gene sets referring to different GO-categories, pathways, diseases, human tissues and specific cell experiments (see methodical section). For each spot we obtained a list of gene sets ranked with increasing *p-*value estimating the probability that genes of the set are found within the spot by chance.

**Figure 3:**
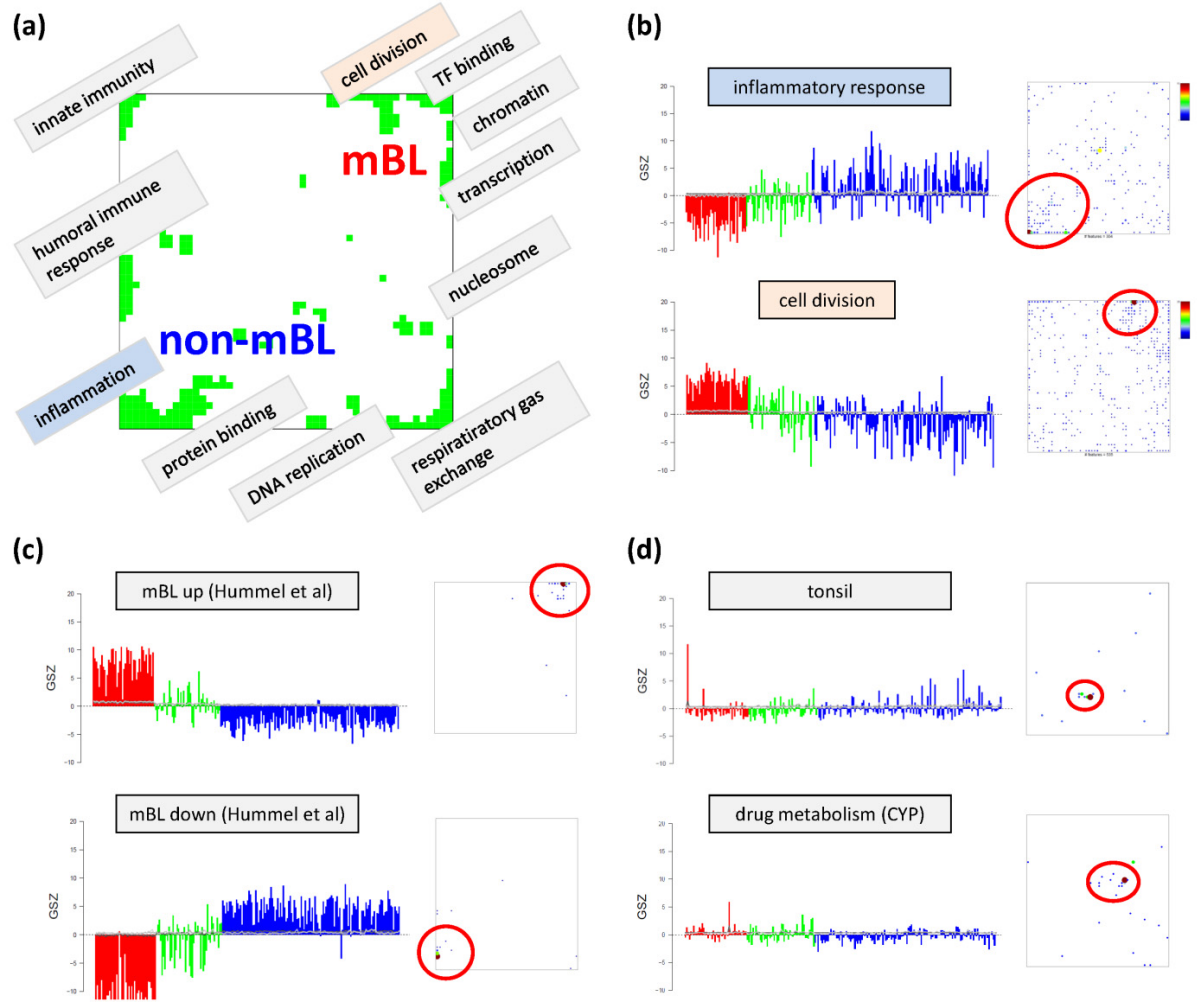
Functional analysis: (**a**) The functional context of the most abundant spots is assigned according to the topmost overexpressed gene sets in each of the spots. (**b**–**d**) GSZ-profiles and population maps are shown for gene sets accumulating in the mBL and non-mBL specific overexpression spots as indicated by the red ellipses (panel b), for mBL-*vs*-non-mBL signature sets published previously [10] (**c**) and for sets accumulating in rare spots (**d**).

Based on the functional context of the overrepresented sets obtained we assign a short notation to each of the spots (see Figure 3a). Some spots are obviously related to processes associated with general hallmarks of cancer such as ‘inflammation’ and ‘cell division’ (spots ‘O’ and ‘K’, respectively). Panel b of Figure 3 depicts the GSZ-expression profiles (left part) and the population maps (right part) of those two leading gene sets. The profiles clearly reflect the fact that the respective processes are selectively over- or under-expressed in a subtype-specific fashion. While ‘inflammatory response’ is activated in the non-mBL subtype, genes annotated to the gene set ‘cell division’ are active in the mBL subtype. The respective gene set population maps reveal that the associated genes accumulate in the regions of spots overexpressed in the respective subtype, as expected.

Neighboring spots of strongly correlated profiles can be assigned to related biological processes: the ‘cell division’ spot is surrounded by spots assigned to ‘transcription factor binding’, ‘chromatin’ and ‘transcription’ according to the most overrepresented gene sets in each of the spots. Note that, although related, these neighboring spots are usually characterized by subtle differences in their expression profiles and presumably also by fine differences in the functional context of the overrepresented gene sets. Population maps and overexpression spot maps therefore represent complementary tools for discovering the functional context of the expression landscapes. The results so far show that the lymphoma samples split into pairs of subtypes differing by the antagonistic activation of processes related to ‘inflammation’ and ‘immune response’ on one hand and to ‘cell division’ and the ‘transcriptional and translational machinery’ on the other hand (non-mBL-*vs*-mBL). 

To validate the subtype-specific spot patterns identified above, we included the signature set that differentiates between mBL and non-mBL subtypes provided by Hummel *et al*. [10] (see Figure 3c). As expected, genes of this set clearly accumulate in the subtype-specific spots ‘L’ and ‘O’ assigned to mBL and non-mBL, respectively.

Another important question is about the possible origin of the rare spots in the central part of the map. In Figure 3d, we show the characteristics of two gene sets related to tissue specific gene expression in tonsils [11,12] and to drug response (‘drug metabolism, cytochrome P450 (CYP)’, see [44]), respectively. Their genes strongly accumulate in localized regions of the map agreeing with the positions of the rare spots ‘S’ and ‘G’, respectively.). Both gene sets are overexpressed in only few samples suggesting that the respective samples are outliers contaminated either with healthy tissue or affected by patient specific medication. Both effects are not related to the cancer studied and thus reflect systematic biases of the respective expression patterns.

### 3.4. Analyzing the Sample Similarity Space

**Figure 4:**
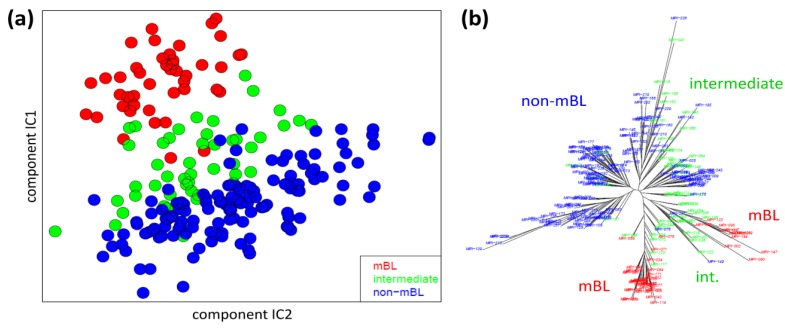
Sample similarity analysis: (**a**) Independent component analysis (ICA) of lymphoma samples. The distribution of the samples is shown in the space spanned by the two leading independent components. (**b**) The neighbor-joining tree projects the sample similarity relations into a dendrogram. The bush-like structures reveal a finer granularity of subtypes beyond the three classes considered so far.

We applied two standard sample similarity analyses, namely independent component analysis (ICA) and neighbor-joining clustering (NJ), to visualize and to analyze the mutual relations between the samples. In the two-dimensional ICA-plot shown in Figure 4a, the samples distribute along the first two components of minimal statistical dependency. It reveals basically three clusters referring to the three subtypes, however without clear boundaries limiting them. It also shows that the three subtypes mainly separate along the IC1-coordinate, whereas intra-subtype variability mainly spreads along the IC2-coordinate.

The NJ algorithm visualizes sample similarity relations as seen by Euclidean distances. The obtained star-like dendrogram shown in Figure 4b identifies ‘bush-like’ clusters containing mostly samples of the same subtype. Interestingly, each of the different subtypes distributes over more than one of such bush-like branches reflecting its intrinsic heterogeneity in terms of disjoint clusters.

In summary, the ICA-analysis allows for estimating the mutual dependence of the expression changes associated with the different subtypes. We found a one-dimensional distribution of the lymphoma subtypes, supporting the ‘longitudinal’ classification into the three subtypes considered so far. The transversal heterogeneity however remains unconsidered in this case. The NJ-dendrogram, on the other hand, reveals finer details in terms of disjunct substructures potentially reflecting a finer granularity of subtype clusters.

### 3.5. Sample Correlation Structure

**Figure 5:**
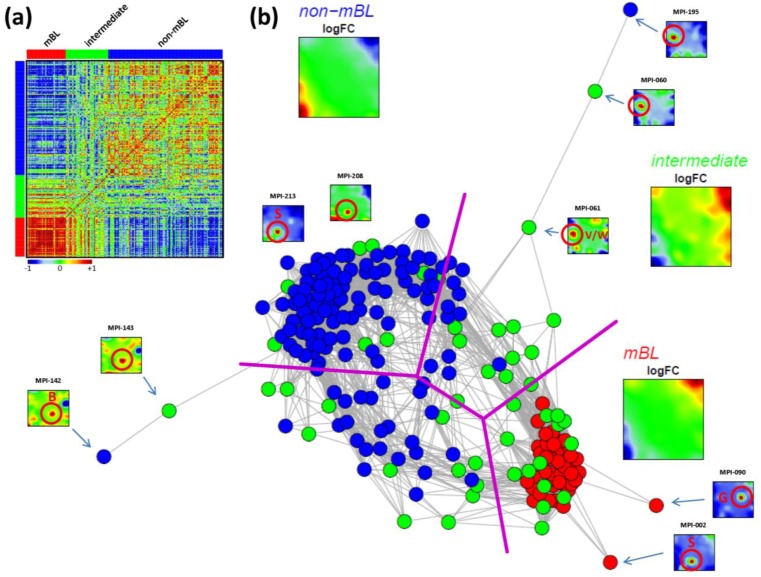
Pairwise correlation analysis of all lymphoma samples: (**a**) The pairwise correlation map (PCM) visualizes the correlation coefficients for all pairs of samples. The samples are arranged according to their subtype membership as indicated by the color bars. In the heatmap, red colors indicate positive, blue colors negative correlations between the samples. (**b**) The correlation network (CN) translates the PCM into a graph structure. The nodes are given by the samples and the edges connect positively correlated sample pairs (*r* > 0.5). Mean subtype portraits are given within the figure (large maps). Outlier nodes are highlighted by arrows. The SOM portraits of the respective samples are shown by small maps. The red circles and the spot letters indicate the outlier spots differing from the subtype specific patterns (compare these individual sample portraits with the mean subtype portraits).

As an alternative metric to study sample similarities, we calculated Pearson’s correlation coefficients for all pairwise combinations of samples. The pairwise correlation map (PCM) given in Figure 5a visualizes the correlation coefficients for all sample pairings which are arranged according to their subtype assignments (see the color bars along the borders of the map). The compact red square of mBL sample couples reflects the strong similarity between their expression landscapes whereas the blue off-diagonal area formed between the mBL and non-mBL samples indicates their anti-correlated expression states. Note that the pairings between non-mBL samples, although correlated, reveal a much more fuzzy pattern due to the more heterogeneous expression states compared to the mBL subtype. The samples of the intermediate subtype either correlate with the mBL or non-mBL samples or with both in some cases.

The correlation matrix can be transformed into the correlation network (CN) shown in Figure 5b. In this graph representation, the samples are represented by nodes connected by edges if the mutual correlation coefficient exceeds a certain threshold. The length of the edges approximately inversely scales with the respective correlation strength. Visual inspection of the CN shows that the mBL and non-mBL samples accumulate into well separated clusters whereas samples of the intermediate subtype heterogeneously spread over the region between these two clusters. Interestingly, these intermediate samples distribute along two disjunctive branches of the CN, which both link the mBL and non-mBL clusters. These two separate branches also include a fraction of the mBL and non-mBL samples (see the purple lines in Figure 5b roughly separating the clusters and branches). This distribution of the intermediate subtype samples reflects the heterogeneous spot characteristics of the subtypes as discussed above.

A few samples are located far away from their subtype-specific cluster and/or from the majority of the other samples in the CN. Those samples are usually characterized by rare or unique spots as indicated in Figure 5b. We will address this issue in the next section more in detail.

In summary, the correlation net of the lymphoma samples forms a ‘donut-like’ structure composed of alternating compact and more fuzzy clusters. The former ones refer to the main subtypes and the latter ones to two distinct groups of samples mainly assigned to the intermediate subtype. The mutual correlation analysis as seen by the CN in combination with the SOM portraits thus provides additional information complementing the other similarity analyses applied.

### 3.6. Detection and Correction of Outliers

Inspection of the CN in Figure 5b reveals a series of samples which are located outside of the main network body. The portraits of these outlier samples reveal overexpression spot patterns deviating from the subtype specific patterns identified in terms of their mean SOM portraits. Particularly the spots ‘G’, ‘S’ and ‘W’ are identified in the outlier sample portraits (red circles in Figure 5b; see Figure 2b for spot-letter assignments). Here, we exemplarily focus on spot ‘S’, located in the bottom-left region of the SOM and strongly overexpressed in samples MPI-002, MPI-208 and MPI-213 (see Figure 5b). The topmost enriched gene set in this spot is the ‘tonsils’-set. It was extracted as the tonsil-signature from a large expression data set of healthy human tissues previously analyzed with our SOM pipeline [11,12]. Enrichment of this set suggests that overexpression of spot ‘S’ is caused by contamination of the tumor biopsy with adjacent healthy lymph node tissue.

**Figure 6:**
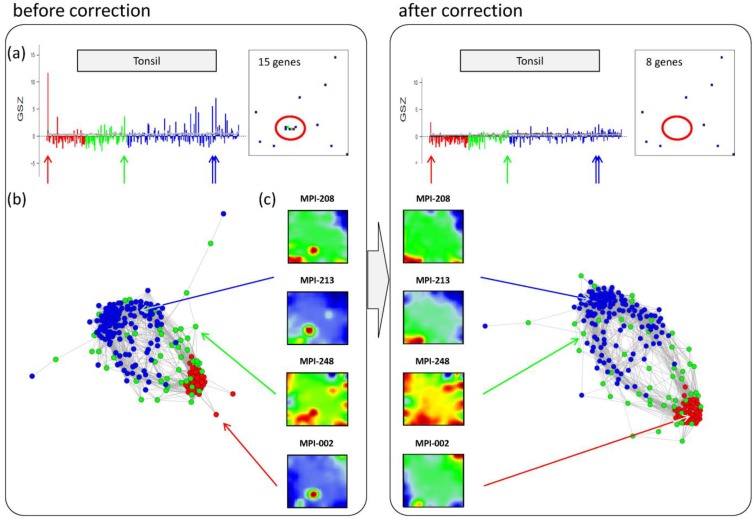
Correction of outlier samples contaminated with healthy lymph node tissue. The left and right parts of the figure refer to the uncorrected and corrected data, respectively. (**a**) GSZ-profile and population map of the ‘tonsil’ gene set: The signature is not characteristic for one of the subtypes and their genes accumulate in spot ‘S’ of the map. (**b**) Correlation network of the lymphoma data set. (**c**) SOM portraits of selected outlier samples. The arrows point to the position of these samples in the CN and in the GSZ-profile. After correction, the expression landscape of the selected samples reveals subtype-specific signatures.

Panel a in the left part of Figure 6 shows the GSZ-profile and the population map of the ‘tonsil’ set. The GSZ-profile reveals very strong overexpression of the set in a number of samples independent of their subtype assignment. The corresponding genes mainly accumulate in spot ‘S’. Selected samples which possess this particular spot in their portraits are shown in Panel c. They can already be identified as potential outliers by simple visual inspection of the SOM portrait gallery (Supplementary File 2). We highlighted the samples in the GSZ-profile (Panel a) and in the CN (Panel b) by arrows. Note however that not all of these samples protrude as clear outliers in the CN. Despite the strong overexpression of the contamination spot ‘S’, the overall expression state of e.g., samples MPI-208 and MPI-213 obviously resemble those of the unbiased samples.

In a simple correction step we removed the genes included in the outlier spots from the whole data set (see red circle in the population maps in Figure 6). This procedure can be repeated for the other contamination spots identified: For example, spot ‘G’ was found to be related to drug metabolism (‘cytochrome p450’, see Figure 3d and sample MPI-090 in Figure 5b), presumably due to individual medication of the patient. Spots ‘V’/’W’ show an intense increase in expression of the G-antigen-family for unknown reasons (samples MPI-060, MPI-061 and MPI-195 in Figure 5b).

After removing strongly biased genes from the training data, we generated a new SOM. Note that, depending on the purpose, also re-evaluation of only parts of the analyses may be sufficient. The right part of Figure 6 shows the results after correction for tonsil-contamination accumulated in spot ‘S’. The corresponding GSZ-profile shows a more uniform expression of the gene set after correction. The respective sample portraits now show the characteristic spot signatures of the respective subtypes, *i.e.*, of mBL for MPI-002 and non-mBL for MPI-208 and MPI-213. Especially the outlier sample MPI-002 is now located within the mBL cluster in the CN, such that it attains a more compact shape.

In summary, the combination of individual portraits, enrichment analysis and the correlation network provides a framework for easy and intuitive detection of outlier spots and samples. After correction, more reliable expression landscapes of the samples are obtained.

### 3.7. Alternative Subtyping of B-Cell Lymphoma

**Figure 7:**
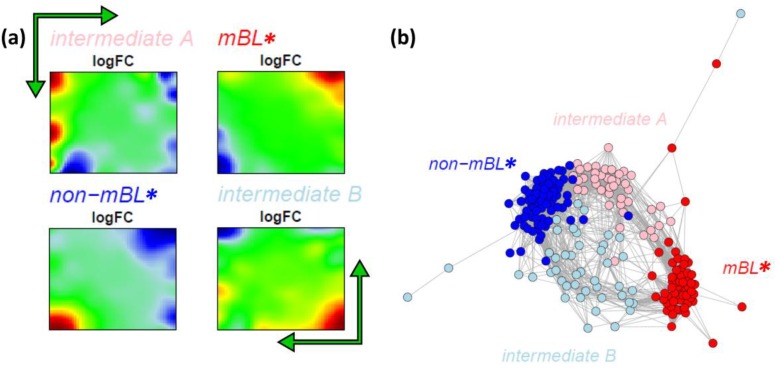
k-Means clustering into four subtypes: (**a**) Mean expression portraits of the four new subtypes. The green arrows indicate the spot pattern transitions from mBL to non-mBL via intermediate A or B. (**b**) CN colored according to the new subtypes obtained.

Our analysis so far suggests that the samples assigned to the intermediate subtype split up into two separate branches which also include samples previously assigned to the mBL and especially the non-mBL subtypes. These two branches are characterized by overexpression spots in the bottom-right and top-left part of the expression portraits, respectively (compare the first and the second row of the intermediate sample portraits in Figure 1). Note that these spot modules are frequently overexpressed in the intermediate-type samples (see spots ‘J’ and ‘Q’, Figure 2a–c). Both, neighbor-joining clustering and correlation network analyses clearly show two distinct sample groups forming two continuous transition ranges linking the compact mBL and non-mBL clusters. These transition ranges include samples of the intermediate and also of the mBL and non-mBL types (Figure 4b and Figure 5b). These results suggest the existence of four subtypes partly differing from the classification into three subtypes discussed so far. In order to further verify this hypothesis, we applied our prototype-guided k-Means algorithm to cluster the samples into four groups (see methods section). The algorithm uses initial prototypes of the expression landscapes which are given by artificial spot patterns referring to the four desired subtypes: spot ‘K’ initializes the new mBL-like subtype mBL*, spot ‘O’ the non-mBL-like subtype non-mBL* and spots ‘J’ and ‘Q’ the two new intermediate subtypes *intermediate A* and *intermediate B*, respectively. Figure 7a shows the obtained four cluster centroids after convergence of the k-Means algorithm. They represent the mean portraits of the four new subtypes mBL*, *intermediate A*, *intermediate B* and non-mBL*. Note that the mean portraits of the mBL* and non-mBL* subtypes closely resample that of the initial mBL and non-mBL classes, respectively (compare with Figure 1). In contrast, the mean portraits of the new *intermediate A* and *intermediate B* subtypes clearly differ from that of the initial intermediate subtype and from that of the mBL* and non-mBL* patterns.

We re-colored the CN plot according to the new subtype classification (Figure 7b). The mBL* and non-mBL* clusters are more compact compared to the initial mBL and non-mBL clusters (compare with Figure 5b). The expression landscapes of the new groups obtained are obviously more homogeneous (see the complete gallery of new assigned sample portraits in Supplementary File 3). The samples of the two intermediate subtypes accurately accumulate along the two separated branches linking the mBL* and non-mBL* clusters except a certain region of overlap in the center of the CN. Further sample similarity analyses based on the four subtype classification support these results (see Supplementary File 1).

In the next step, we compare the robustness of the old and new subtype cluster assignments by applying the bootstrap clustering approach described in the methods section. It returns the bootstrap stability score for each sample in the range of [0, 1] for unstable to very stable assignments. For the previous classification into three subtypes, the stability scores of the intermediate and mBL subtype samples show a broad distribution with scores of 0.5 and below (see Supplementary File 1 for details). The new four subtype classification is clearly more robust, reflecting a more consistent and stable clustering of the samples. Only a small number of relatively uncertainly assigned samples are found even in the transition ranges between the different clusters.

### 3.8. Consensus Clustering of B-Cell Lymphoma

**Figure 8:**
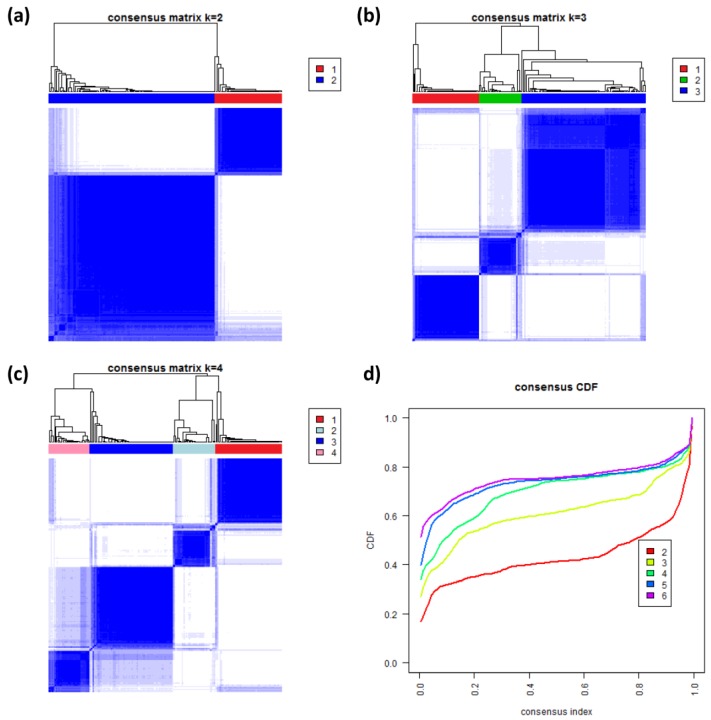
Consensus clustering: (**a**–**c**) Cluster-heatmaps of the consensus matrices for class numbers ranging from two to four, respectively. Pairs of samples frequently found in one joint class accumulate in the blue regions along the diagonal of the map. (**d**) Cumulative distribution function (CDF) for class numbers ranging from two to six.

To further validate our new subtypes we applied consensus clustering to estimate the optimal number of classes in the lymphoma data by an independent method which assumes class numbers *k* ranging from two to six. Figure 8a–c shows the heatmaps of the consensus matrix for two to four classes, respectively. Pairs of samples, robustly assigned to the same cluster, accumulate within one of the blue squares along the diagonal of the heatmap. The two-class approach basically divides the samples into an mBL-like and a non-mBL-like cluster (Figure 8a). The three-class approach essentially splits the samples into the mBL/intermediate/non-mBL subtype structure as proposed in [10] (Figure 8b). The four-class consensus clustering resembles our new subtype classification with the two intermediate subtypes (Figure 8c). The five- and six-cluster approaches virtually do not change this result: the additional fifth and sixth clusters collect only one and three outlier samples, respectively (data not shown).

The cumulative distribution functions (CDFs) allow judging the incremental gain of increasing the number of clusters (see Figure 8d). The obtained CDFs support the four-class approach: the CDF converge for *k* > 3 showing only small incremental changes with further increasing *k*. Note that the increment between *k* = 4 and 5 is caused by a single-sample cluster. Hence, consensus clustering confirms our four-subtype classification.

### 3.9. Functional, Molecular and Phenotypic Characterization of the New Subtypes

The four new subtypes are defined by their distinct expression patterns and their particular functional contexts, *i.e.*, they represent molecular subtypes. The question arises if these molecular subtypes associate with selected genetic, clinical, or alternative molecular phenotypes collected independently [10]. We used these data and calculated the frequency distribution of patients for each of the characteristics over the four subtypes. Table 1 reveals associations between these characteristics and the subtypes in terms of enriched or depleted patient numbers (*p-*values are obtained from Fisher’s exact test). The full table of patient characteristics is provided as Supplementary File 4.

**Table 1:** Phenotypic and molecular characterization of the four new subtypes.

Characteristic ^a^			Lymphoma subtype				*p*-value ^b^
			mBL*	*intermediate A*	*intermediate B*	*non-mBL* *	
**Total**	number of patients	221	62 (28%)	42 (19%)	44 (20%)	73 (33%)	
**Age**	<20 y	32 (14%)	**26 (42%)**	0 (0%)	1 (2%)	5 (7%)	<0.001
	21–65 y	92 (42%)	27 (44%)	14 (33%)	22 (50%)	29 (40%)	
	>66 y	95 (43%)	9 (15%)	27 (64%)	20 (45%)	39 (53%)	
**Gender**	male	127 (57%)	40 (65%)	26 (62%)	23 (52%)	38 (52%)	0.44
	female	91 (41%)	22 (35%)	15 (36%)	20 (45%)	34 (47%)	
**Diagnosis**	Burkitt's lymphoma	15 (7%)	**15 (24%)**	0 (0%)	0 (0%)	0 (0%)	<0.001
	Atypical Burkitt’s lymphoma	20 (9%)	16 (26%)	3 (7%)	0 (0%)	1 (1%)	
	Diffuse large-B-cell lymphoma	164 (74%)	24 (39%)	**37 (88%)**	**38 (86%)**	**65 (89%)**	
	Mature aggressive B-cell lymphoma, unclassifiable	18 (8%)	5 (8%)	2 (5%)	5 (11%)	6 (8%)	
**Ann Arbor stage**	I or II	72 (33%)	25 (40%)	9 (21%)	15 (34%)	23 (32%)	0.37
	III or IV	82 (37%)	19 (31%)	15 (36%)	22 (50%)	26 (36%)	
**Response to treatment**	Complete remission	68 (31%)	27 (44%)	8 (19%)	10 (23%)	23 (32%)	0.40
	Complete remission, unconfirmed	18 (8%)	4 (6%)	2 (5%)	6 (14%)	6 (8%)	
	No change	2 (1%)	0 (0%)	0 (0%)	1 (2%)	1 (1%)	
	Partial response	16 (7%)	1 (2%)	3 (7%)	5 (11%)	7 (10%)	
	Progress	24 (11%)	7 (11%)	4 (10%)	7 (16%)	6 (8%)	
**Molecular classification**	mBL	44 (20%)	**44 (71%)**	0 (0%)	0 (0%)	0 (0%)	<0.001
Hummel *et al*. [10]	intermediate	48 (22%)	18 (29%)	11 (26%)	10 (23%)	9 (12%)	
	non-mBL	129 (58%)	0 (0%)	**31 (74%)**	**34 (77%)**	**64 (88%)**	
**GCB-ABC classification**	Activated B-cells	58 (26%)	2 (3%)	**26 (62%)**	15 (34%)	15 (21%)	<0.001
Wright *et al*. [45]	Germinal center B-cells	120 (54%)	53 (85%)	10 (24%)	18 (41%)	39 (53%)	
	unclassified	43 (19%)	7 (11%)	6 (14%)	11 (25%)	19 (26%)	
**Translocations**							
MYC translocation	IG-MYC	60 (27%)	49 (79%)	1 (2%)	**6 (14%)**	4 (5%)	<0.001
	non-IG-MYC	15 (7%)	6 (10%)	5 (12%)	2 (5%)	2 (3%)	
	neg	144 (65%)	7 (11%)	36 (86%)	35 (80%)	66 (90%)	
BCL6 Break	pos	37 (17%)	2 (3%)	9 (21%)	11 (25%)	15 (21%)	0.002
	neg	179 (81%)	59 (95%)	32 (76%)	31 (70%)	57 (78%)	
IGH Break	pos	115 (52%)	53 (85%)	**11 (26%)**	23 (52%)	28 (38%)	<0.001
	neg	103 (47%)	9 (15%)	30 (71%)	20 (45%)	44 (60%)	
t(14;18) translocation	pos	25 (11%)	5 (8%)	2 (5%)	**6 (14%)**	**12 (16%)**	0.19
	neg	193 (87%)	57 (92%)	40 (95%)	37 (84%)	59 (81%)	
**Immunohisto-chemistry**							
CD10	low	114 (52%)	3 (5%)	33 (79%)	26 (59%)	52 (71%)	<0.001
	high	96 (43%)	**56 (90%)**	6 (14%)	14 (32%)	20 (27%)	
BCL2	low	62 (28%)	38 (61%)	**2 (5%)**	7 (16%)	15 (21%)	<0.001
	high	153 (69%)	22 (35%)	39 (93%)	35 (80%)	57 (78%)	
BCL6	low	34 (15%)	5 (8%)	9 (21%)	7 (16%)	13 (18%)	0.21
	high	168 (76%)	52 (84%)	29 (69%)	32 (73%)	55 (75%)	
MUM1	low	66 (30%)	29 (47%)	**7 (17%)**	**8 (18%)**	22 (30%)	0.001
	high	139 (63%)	27 (44%)	33 (79%)	32 (73%)	47 (64%)	
KI67	low	125 (57%)	17 (27%)	26 (62%)	26 (59%)	56 (77%)	<0.001
	high	89 (40%)	**44 (71%)**	15 (36%)	14 (32%)	16 (22%)	

^a^ Percentages refer to the total number of samples. Parameters are not available for all samples. Data are taken from ref [43]; ^b^ *p-*values are calculated using Fisher’s exact test.

For mBL* and non-mBL* one finds analogous frequency distributions of a series of characteristics as described in previous studies, e.g., the age dependency [10], the effect of the MYC-gene translocation [10], different immune-phenotypes [46] and the GCB-ABC-signature [45]. Nearly 90% of the lymphoma assigned to the non-mBL* and to *intermediate A&B* subtypes are classified as diffused, large B-cell lymphoma (DLBCL) suggesting a close similarity between these three subtypes. A series of characteristics such as the IG-MYC status and immune-phenotypes CD10, BCL6 and BCL2 support this result. 

However, the new *intermediate A* and *intermediate B* subtypes also show specific properties. Interestingly, the tumors with the activated B-cell (ABC) signature are clearly overrepresented in the *intermediate A* subtype, whereas the alternative germinal center B-cell (GCB) signature clearly depletes in this subtype. They also show differential characteristics with respect to the appearance of genetic aberrations (MYC translocation and IGH break) and to the BCL2 immune-phenotype: Firstly, the IG-MYC translocation is more frequently found in the *intermediate B* subtype compared with the *intermediate A* and the non-mBL* lymphoma. Secondly, *intermediate A* lymphomas less frequently show the IGH break and the BCL2+ immuno-phenotype than the other subtypes. Thirdly, *intermediate B* and non-mBL* lymphomas possess slightly enriched populations of t(14;18)(q32;q21) translocations, which juxtapose the BCL2 oncogene to the immunoglobulin heavy chain locus (IGH).

In the supplementary text (Supplementary File 1), we provide a thorough analysis of the expression signatures of the subtypes, the co-expression network of the spot modules and their functional impact. It turned out that each of the subtypes is characterized by different hallmarks of cancer, e.g., proliferation and high transcriptional and translational activity in mBL*; activated immune response and inflammation in non-mBL*, innate immunity in the *intermediate A* subtype and up-regulated expression of common cancer gene signatures [47] in the *intermediate B* subtype. Generic, MYC-related poor prognosis gene signatures [48] are associated with the mBL* and, to a lesser degree, *intermediate A* subtypes. Moreover, we found that *intermediate A* subtype lymphomas show expression signatures of activated B-cells and strong dissimilarity with expression landscapes of germinal center B-cells and healthy lymph node tissue suggesting different cell-of-origins. On the level of gene regulation, the decomposition of lymphoma into four subtypes obviously further diversifies into different modes which, in turn, reflect driving effects on the genetic and epigenetic levels. The understanding of these molecular mechanisms thus requires the combined analysis of genetic, epigenetic and transcriptional data. 

**Figure 9:**
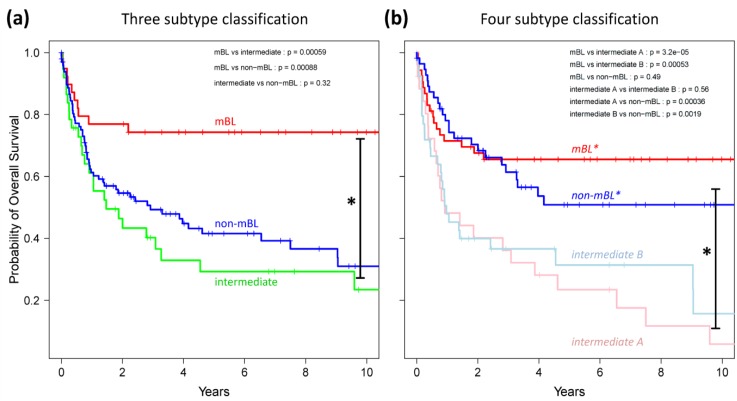
Kaplan-Meier survival curves of the original three subtypes (**a**) and the new four subtype (**b**) classifications. Tick marks indicate patients alive at the time of last follow-up. Subtype specific survival curves are compared using log-rank test and the respective *p-*values are indicated within the figures.

Finally, we generated Kaplan-Meier diagrams to estimate the probability of subtype specific overall patient survival as a function of time [49]. Figure 9a,b show the curves for the three and four subtype classifications, respectively. Based on the original definition by Hummel *et al*., patients with mBL lymphomas show significantly better survival rates as intermediate and non-mBL patients (*p* < 0.001 in log-rank test, see also [10]). In contrast, our new classification now reveals that both mBL* and non-mBL* patients show better survival rates than patients of the *intermediate A & B* subtypes. Assignment of lymphoma to either of the two intermediate subtypes roughly halves the survival rate. The diversification of lymphoma subtypes thus clearly impacts prognosis.

A recent study also proposed new classes of B-cell lymphoma based on a correlation gene set analysis and using a larger patient collective [42]. This study excluded mBL samples from the patient cohort and divided the remaining diffuse large B-cell cases into three classes. Their expression signatures and phenotypic characteristics show certain similarities with our non-mBL*, *intermediate A* and *B* subtypes; however, they also differ in other properties, for example in the assignment of cell-of-origin properties and of energy metabolism signatures.

## 4. Conclusions

Analysis of molecular biological data using Self-Organizing Maps (SOMs) enables a holistic view on high-dimensional data collected in large-scale studies. It provides a general framework for analytic tasks such as feature selection, integration of concepts of molecular function and systems tracking with individual resolution. The method extracts meta-features such as metagenes and spot-modules representing basal modes of systems behavior important for higher-level analysis. 

We applied SOM machine learning to patient expression data of mature aggressive B-cell lymphomas to characterize the specifics of the genome wide expression landscapes in different molecular subtypes of lymphoma. The expression portraits obtained by the SOM algorithm reflect the expression landscapes of the individual samples or subtypes in terms of intuitive and characteristic color textures. These spot patterns can be used to describe the underlying functional modules using gene set enrichment techniques.

Several sample similarity analysis methods were applied to characterize the subtype structure in detail. The correlation network approach provides a powerful representation as it visualizes multivariate relationships in a clear and accessible fashion. We presented a straightforward strategy to identify outlier samples and modules, e.g., due to contaminations of tumor samples with healthy tissue, and to correct them. Furthermore, we found indications for a finer subtype classification of aggressive B-cell lymphoma into four subtypes. Samples were classified using a spot-guided and metagene-based k-Means clustering method. The robustness and consensus-cluster stability of the new four subtypes exceeds that of previous three class approaches. The functional and clinical impact of the new subtypes was discussed. The two *intermediate* subtypes of heterogeneous molecular signatures are associated with poor survival prognosis compared with the more homogeneous mBL* and non-mBL* subtypes.

Our case study shows that analyzing gene expression landscapes with the tools presented here facilitates information mining in such huge data sets and eventually promotes our understanding of cancer biology.

## Supplementary Files

Supplementary File 1 Supplementary (ZIP, 8169 KB)

## Supplementary Materials

Supplementary File 1: Supplementary text.

Supplementary File 2: Complete gallery of all 221 sample expression portraits.

Supplementary File 3: Complete gallery of all 221 sample expression portraits using the new classification into four subtypes.

Supplementary File 4: Clinical characteristics of the patients arranged according to three and four subtype classifications.
