# Supplementary material for: Portraying the Expression Landscapes of B-Cell Lymphoma-Intuitive Detection of Outlier Samples and of Molecular Subtypes
Source: Biology (Basel). 2013 Dec 2;2(4):1411–37. doi: 10.3390/biology2041411 (PMC4009791; doi:10.3390/biology2041411)
Supplement: Supplementary File 1 — Supplementary (ZIP, 8169 KB) [file biology-02-01411-s001.zip › supplementary/additional file 01-proofread.pdf]

Biology **2013**, *2*, 1-x manuscripts; doi:10.3390/biology20x000x

OPEN ACCESS

biology

ISSN 2079-7737

www.mdpi.com/journal/biology

Article

Portraying the Expression Landscapes of B-Cell Lymphoma-Intuitive Detection of Outlier Samples and of Molecular Subtypes

Lydia Hopp [1,2], Kathrin Lembcke [1], Hans Binder [1] and Henry Wirth [1,2,*]

[1] Interdisciplinary Centre for Bioinformatics, Universität Leipzig, Härtelstr. 16–18, Leipzig 04107, Germany; E-Mails: hopp@izbi.uni-leipzig.de (L.H.); lembcke@izbi.uni-leipzig.de (K.L.); binder@izbi.uni-leipzig.de (H.B.)

[2] LIFE, Leipzig Research Center for Civilization Diseases, Universität Leipzig, Philipp-Rosenthal-Straße 27, Leipzig 04103, Germany

* Author to whom correspondence should be addressed; E-Mail: wirth@izbi.uni-leipzig.de; Tel.: +49-341-9716697; Fax: +49-341-9716669.

Received: 1 August 2013; in revised form: 1 October 2013 / Accepted: 5 November 2013 / Published: 25 November 2013

Supplementary Materials

1. Supporting Maps and Profiles

A series of so-called supporting maps and profiles provide additional information about the metagenes beyond the expression portraits (see [1,2] for detailed descriptions). The population map visualizes the number of single genes mapped to each metagene. It shows that the single genes heterogeneously distribute among the map (Figure S1a). The metagene of maximal population (n_k = 962, see the dark red tile in the center of the map) refers to genes with virtually invariant, mostly absent (*i.e.*, below the detection limit) expression values in all samples studied. These invariant genes give rise to the dark blue spot area in the center of the variance map (Figure S1b). The population map also reveals so-called ‘empty’ metagenes in the central and top left regions of the map. Note that downstream analyses such as gene lists and gene set enrichment analysis use the single gene information behind the metagenes. Empty metagenes thus do not contribute to these results. They effectively serve as a sort of separator between different expression modules characterized by different profiles. Note that all metagenes are characterized by their expression profiles which are adapted to optimally cover the input data space in the training process [3]. Empty metagenes do not interfere with the training process: There, the association of genes to metagenes changes continuously and adaption of a metagene profile also affects neighboring metagenes.

Figure S1. The population map illustrates the number of single genes mapped to each metagene: (**a**) The variance map illustrates the variance of the metagene expression profiles in each of the tiles (**b**); The variance of the expression state of each sample and of each subtype (**c**) and the distribution of spot numbers in each of the subtypes (**d**) are shown for the three subtype and four subtype classifications, respectively.

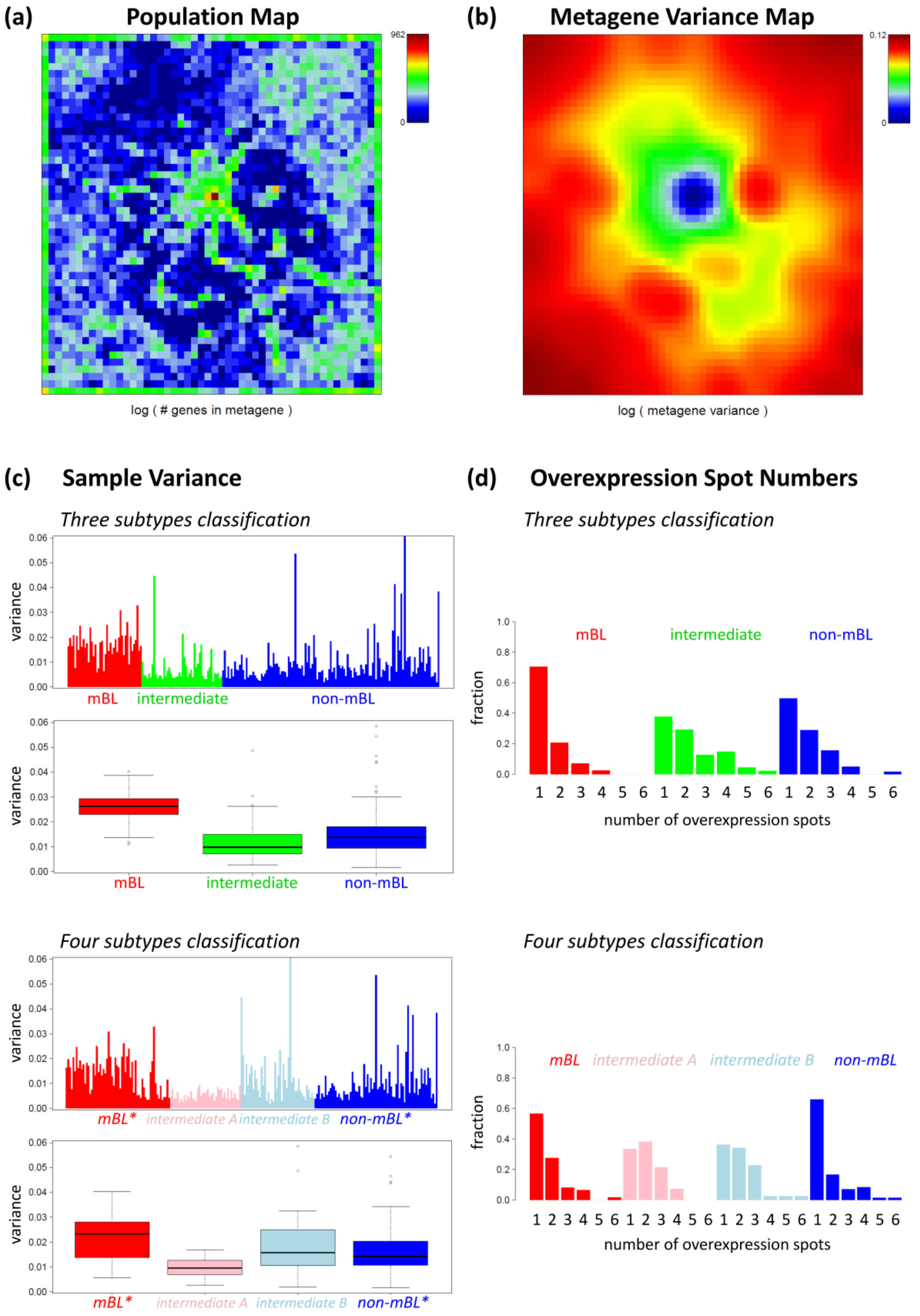

The variance profiles provide the variability of the metagene expression landscape of each sample. They are shown as single sample barplots and as culminated boxplots for each subtype of the three and the four subtype classifications (Figure S1c). They reveal, for example, a significantly higher variability of the mBL subtype samples compared with the intermediate and non-mBL ones ($p < 0.0001$ in Wilcoxon rank-sum test), as well as a significantly lower variability of the *intermediate A* subtype ($p < 0.0001$). In other words, samples of the *intermediate A* subtype possess a more flat expression landscape than the *intermediate B* and *non-mBL** subtypes, whereas the landscapes of the *mBL** samples are the most 'mountainous' ones.

Finally, the distributions of the numbers of overexpression spots in the samples are given for the original and the new subtypes. They reveal that sample portraits of the subtypes mBL and non-mBL, as well as *mBL** and *non-mBL** show only one spot in most cases, whereas the different intermediate subtypes show a broader distribution of spot numbers (Figure S1d). Especially, the *intermediate B* and even to a larger extent the *intermediate A* subtypes are relatively heterogeneous with respect to the number of spots and, thus, number of different expression modules.

2. Correlation Network Representation

The correlation network (CN) translates the pairwise sample-sample correlation matrix into one graph. Its edges connect pairs of samples whose pairwise correlation coefficient exceeds a defined threshold. We chose $r_{threshold} = 0.5$ as default value (Figure S2b), which was successfully applied in previous cancer studies [4]. It was chosen as the most stringent threshold which still provides one connected graph. Higher correlation thresholds lead to isolated nodes (Figure S2c), whereas lower thresholds give rise to more dense graphs with partly unresolved structure (Figure S2a).

Figure S2. The correlation network of lymphoma is generated using differing correlation thresholds: $r_{threshold} = 0.4$ (**a**), $r_{threshold} = 0.5$ (**b**) and $r_{threshold} = 0.6$ (**c**). We chose the threshold 0.5 as default value to optimally resolve the network structure and to avoid isolated nodes.

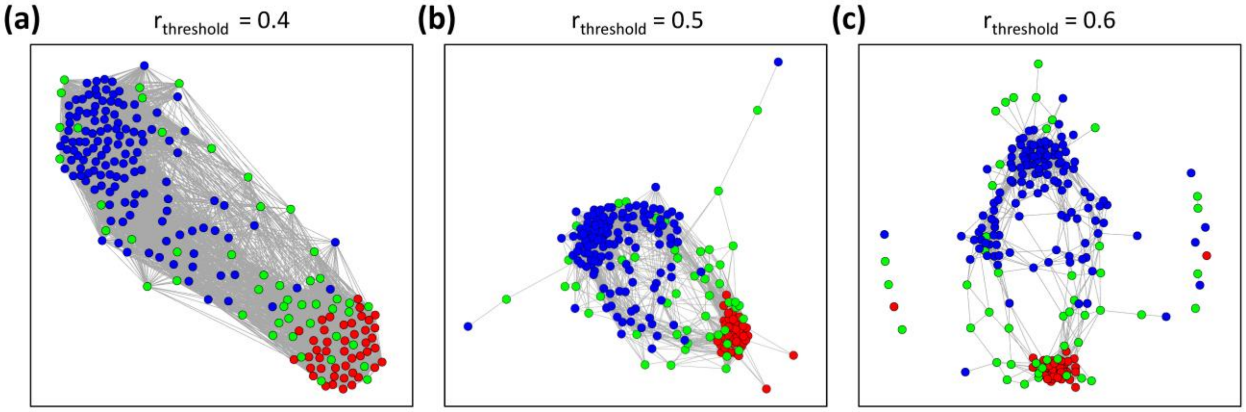

3. Sample Similarities Analysis of the New Subtypes

The sample similarity analyses described in the manuscript are performed also for the four new subtypes proposed. The ICA plot reveals four consistent and well defined clusters (Figure S3a,b). The new *mBL** and *non-mBL** subtypes clearly split along the first independent component IC1 whereas

both intermediate subtypes separate along the IC2 coordinate. This result reveals that *mBL** and *non-mBL** subtypes on one hand and *intermediate A & B* on the other hand are governed by two mutually independently changing groups of genes. Especially, the *non-mBL** samples extend also along the IC3 component reflecting the relatively heterogeneous character of the subtype.

The NJ-tree shows that the new *intermediate A* and *intermediate B* subtypes accumulate in two disjunct branches as expected (Figure S3c). Interestingly, samples of the *intermediate A* subtype are also found in the *mBL**- and in one of three *non-mBL**-branches supporting its intermediate characteristics in between these subtypes.

Finally, the PCM reveals the intermediate character of both *intermediate* subtypes showing positive and negative correlations with the *mBL** and *non-mBL** samples as well. On the other hand, the two *intermediate* subtypes (and also the *mBL** and *non-mBL**) tend to be pairwise anti-correlated indicating the partly antagonistic character of their gene expression patterns. Note that the four subtype classification provides clearly better structured correlation patterns compared with the three subtype classifications (Figure S3d, compare with Figure 5 in the main paper).

Figure S3. Sample similarity analysis of lymphoma samples using four subtypes: ICA results for the first two (**a**) and first three (**b**) independent components, neighbor-joining tree (**c**) and pairwise correlation matrix (**d**).

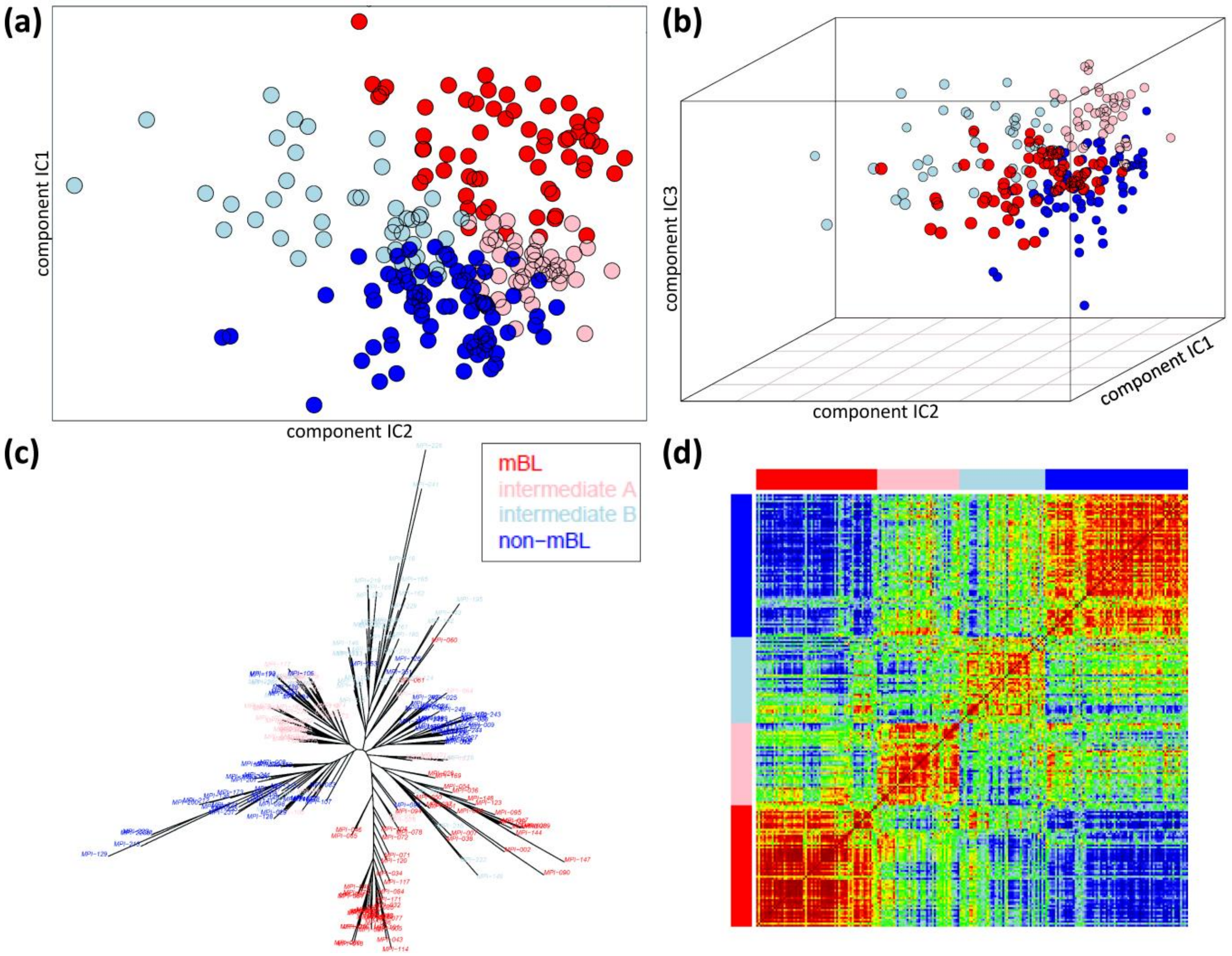

4. Bootstrapping of the Three and Four Subtype Classifications

We apply a bootstrapping approach to compare robustness of the three and four subtype classifications. The robustness is calculated as bootstrap stability score for each sample providing a value in the range of [0, 1]. For the original classification into three subtypes, the stability scores of the intermediate and especially of the non-mBL subtype samples are very diverse ranging from values near unity down to values below 0.5 (Figure S4, upper part). In this respect, the new classification into four subtypes is clearly more robust ($p < 0.0001$, Wilcoxon signed-rank test), reflecting the much more consistent and stable clustering of the samples.

Figure S4. Bootstrap stability scores (upper part), correlation networks and subtype specific mean expression portraits (lower part) are shown for three (mBL, intermediate and non-mBL) and four subtype (*mBL**, *intermediate A*, *intermediate B* & *non-mBL**) classifications.

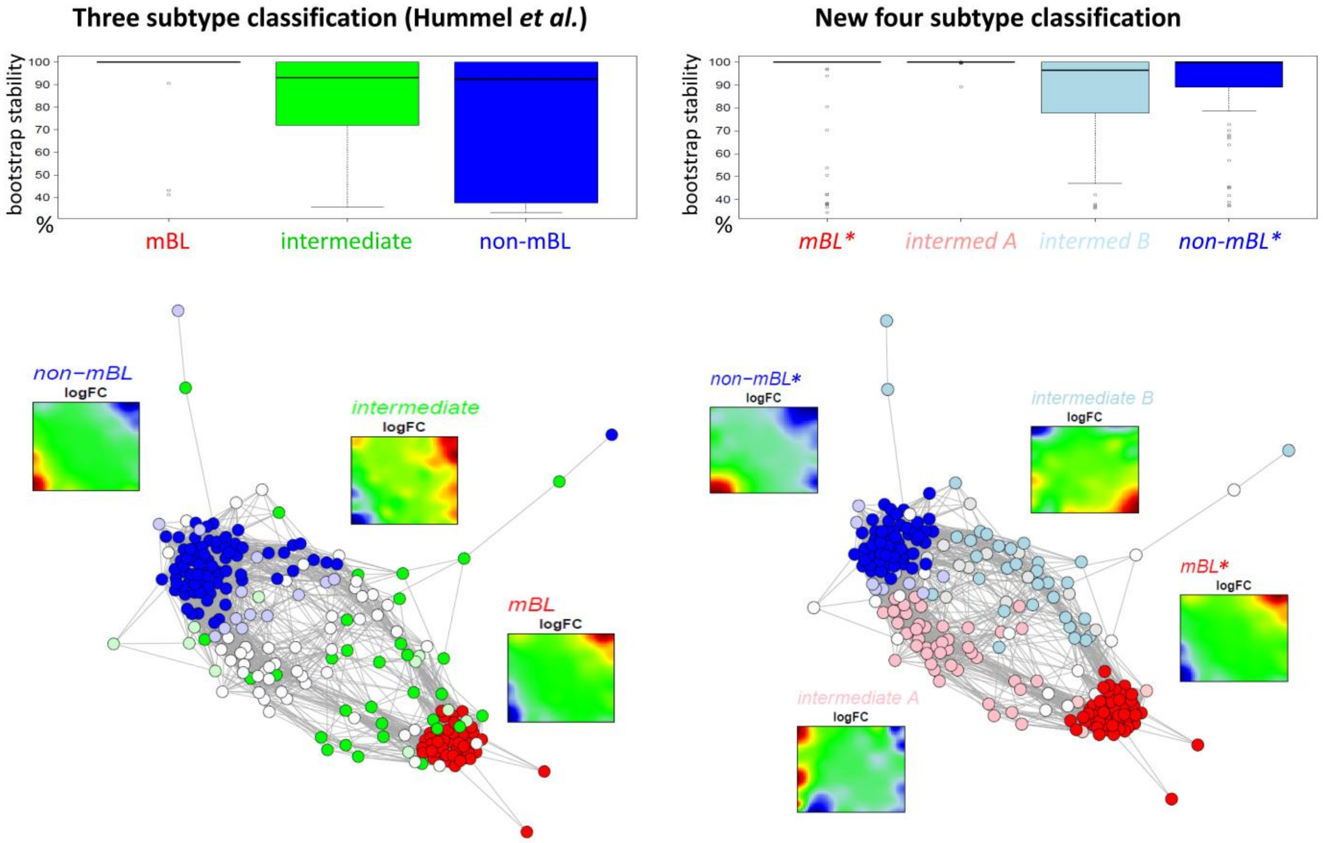

The CNs are redrawn for the two different subtype classifications to localize uncertainly assigned samples which are represented as brightened up or blanked out circles for stability scores below 0.8 and 0.5, respectively (Figure S4, lower part). The CN of the three class approach reveals cluster cores formed by the most stable samples, surrounded by a layer of uncertainly assigned samples. In the four class approach, the number of uncertain samples is clearly smaller. Again they accumulate along the borders between the subtypes. It is noteworthy that the core samples of the mBL and *mBL** (and of the non-mBL and *non-mBL**) clusters in both subtype approaches well agree.

5. Characterization of the New Subtypes

5.1. Modular Gene Regulation Characterizes the Subtypes

To achieve a more general view on the gene expression patterns in the lymphoma subtypes, we applied the weighted topological overlap network (wTO, [5]) approach to the spot modules identified in the lymphoma SOM as described in Hopp *et al*. [4]. The topological overlap ensures that strongly overlapping interactions (*i.e.*, if two spots are strongly correlated with a third one) contribute with stronger weightings than weak ones (e.g., if at least one of the spots poorly correlates with the third one). The topological overlap consequently takes into account direct and also indirect relations between the spot modules. The resulting wTO network of B-cell lymphoma is depicted in Figure S5. Functional assignment of the spots is taken from gene set enrichment analysis. The detailed results of the spot analyses are given in Table S1. In addition, we show the expression profiles and population maps of selected gene sets in Figure S6.

Figure S5. Network of the spot modules calculated using the wTO-metrics: The letters assign the spots as defined in the main paper. Red and blue lines refer to negative and positive correlations, respectively. Strongly correlated signature spots of the same subtype were aggregated into one module (e.g., ‘H’, ‘K’, ‘L’ and ‘M’ for *mBL**). The leading functional context was taken from gene set enrichment analysis.

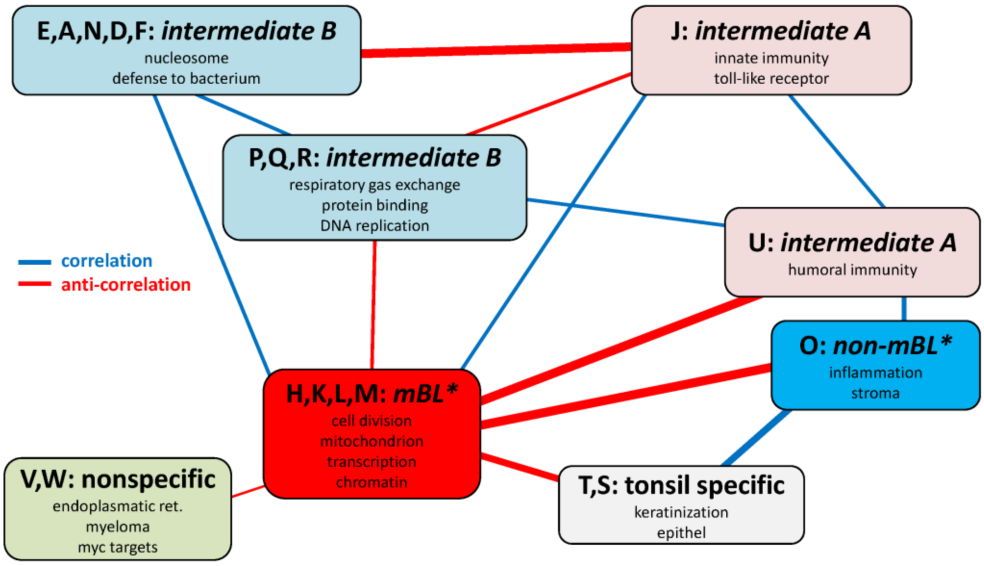

The wTO network reflects the antagonistic relations between spots ‘H’, ‘K’, ‘M’ and ‘L’ up-regulated in *mBL** subtype and spot ‘O’ up-regulated in the *non-mBL** subtype (see Figure 2 in the main paper for spot assignments). The respective genes are associated with hallmarks of cancer. In the mBL/*mBL** subtypes, proliferative and metabolic processes and GO-terms related to ‘cell division’, to transcription and translation (e.g., ‘translation’, ‘nucleus’, ‘ribosome’) and to energy metabolism (e.g., ‘mitochondrial respiratory chain’, ‘mitochondrial outer membrane’) become activated (spots ‘H’, ‘K’, ‘L’ and ‘M’). Against this, samples of the non-mBL/*non-mBL** subtypes up-regulate inflammatory processes and related GO terms such as ‘immune response’ and ‘extracellular space’ (spot ‘O’) (see also Figure S6).

Table S1. Top-enriched gene sets in spots (see Figure 2 in main paper for assignments).

Spot [a]	Short name	#G/#MG [b]	Up [c]	Down [c]	GO and pathway sets [d]	Tissue and disease sets [e]
H	transcript-tion	308/14	*mBL**	*non-mBL**	chromatin silencing (BP, -4), synaptonemal complex (CC, -5); histone deacetylase binding (MF, 4); regulation of transcription, DNA-dependent (BG,-5)	HUTTMANN_B_CLL_POOR_SURVIVAL_DN (LS, -7); MULLIGHAN_MLL_SIGNATURE_2_DN (LS, -7); ZHAN_LATE_DIFFERENTIATION_GENES_DN (LS, -8)
K	cell division	531/26	*mBL**	*non-mBL**	cell division (BP,-17); mitosis (BP, -16), chromosome (CC, -16); ATP binding (MF, -7); nucleus (CC, -17)	SOTIRIOU_BREAST_CANCER_GRADE_1_VS_3_UP (LS, -9); RHODES_UNDIFFERENTIATED_ CANCER (LS, -7); Overlap genes (LS, -7); KANG_DOXORUBICIN_RESISTANCE_UP (LS, -14); MMML 15 (LS, -5), developing astrocytes (CS, -11)
L	TF Binding	323/14	*mBL**	*non-mBL**	nucleus (CC, -7), transcription factor binding (MF, -5); heterochromatin (CC, -4); negative regulation of phosphorylation (BP, -4); histone H3 acetylation (BP, -4); negative regulation of NF-kappaB transcription factor activity (BP, -4)	HUMMEL_BURKITTS_LYMPHOMA_UP (LS, -16); BILBAN_B_CLL_LPL_UP (LS, -6), SCHWAB_TARGETS_OF_BMYB_S427G_UP (LS, -6); SCHWAB_TARGETS_OF_BMYB_I624M_UP (LS, -6); BUCKANOVICH_T_LYMPHOCYTE_ HOMING_ON_TUMOR_DN (LS, -5)
M	chromatin	120/3	*mBL**	*non-mBL**	chromatin modification (BP, -5), nucleus (CC, -5); RNA helicase activity (MF, -5); ribosome binding (MF, -4)	CHEOK_RESPONSE_TO_HD_MTX_DN (LS, -6),
N	nucleo-some	87/2	*mBL**	*non-mBL** *intermediate*	nucleosome assembly (BP, -7), nucleosome (CC, -8); flavin adenine dinucleotide binding (MF, -4); APOPTOSIS_INDUCED_DNA_FRAGMENTA TION (RE, -6)	SCIAN_INVERSED_TARGETS_OF_TP53_AND_TP73_UP (LS, -6); NICK_RESPONSE_TO_PROC_TREATMENT_UP (LS, -6)
J	toll-like receptor (innate immunity)	362/15	*intermediate A*		Toll-like receptor 1–4 signaling pathway (BP, -9); cytosol (CC, -10); protein binding (MF, -10); stress-activated MAPK cascade (BP, -5); Ras protein signal transduction (BP,-5); innate immune response (BP, -4); TOLL_RECEPTOR_CASCADES (RE, -4)	TURJANSKI_MAPK8_AND_MAPK9_TARGETS (LS, -9); TURJANSKI_MAPK14_TARGETS (LS, -6); B-cells (TF, -4); B-cells (TS, -4); GILMORE_CORE_NFKB_PATHWAY (LS, -4);

Table S1. *Cont.*

Spot [a]	Short name	#G/#MG [b]	Up [c]	Down [c]	GO and pathway sets [d]	Tissue and disease sets [e]
U	humoral immune response	321/20	*intermediate A* *non-mBL**	*mBL**	bone resorption (BP, -5), SH3/SH2 adaptor activity (MF, -8); humoral immune response (BP, -5); B cell differentiation(BP, -4)	ROSS_LEUKEMIA_WITH_MLL_FUSIONS (LS, -6), TSUDA_ALVEOLAR_SOFT_PART_SARCOMA (LS, -6), miRNA-503 (LS, -4); SHIPP_DLBCL_CURED_VS_FATAL_DN (LS, -6)
E		71/4	*intermediate B*		defense response to bacterium (BP, -4), basement membrane (CC, -5); ligand-dependent nuclear receptor activity (MF, -4)	TRAYNOR_RETT_SYNDROM_UP (LS, -5); BUSA_SAM68_TARGETS_UP (LS, -4)
P	protein binding	74/6	*intermediate B* *non-mBL**	*mBL**	regulation of protein binding (BP, -7); plasma membrane (CC, -7); elevation of cytosolic calcium ion concentration (BP, -7); membrane depolarization (BP, -6)	CROONQUIST_NRAS_SIGNALING_UP (LS, -6); LIU_NASOPHARYNGEAL_CARCINOMA (LS, -5)
Q	respiratory gas exchange	803/30	*intermediate B*		Respiratory gas exchange (BP, -4); activation of JUN kinase activity (BP, -4); neural crest cell migration (BP, -4); RNA polymerase II core promoter proximal region sequence specific DNA binding transcription factor a (MF, -4)	LIU_COMMON_CANCER_GENES (LS, -7); SPIRA_SMOKERS_LUNG_CANCER_DN (LS, -10); TIAN_TNF_SIGNALING_NOT_VIA_NFKB (LS, -5); GAL_LEUKEMIC_STEM_CELL_U (LS, -5); AGIV_CD24_TARGETS_UP/DN (LS, -5); PIK3_DN (LS, -5)
O	inflamma-tion	1088/76	*non-mBL**	*mBL**	immune response (BP, -16), proteinaceous extracellular matrix (CC, -14); extracellular matrix structural constituent (MF, -13); THELPER_PATHWAY (BC, -8); signal transduction (BP, -11)	HUMMEL_BURKITTS_LYMPHOMA_DN (LS, -12); KOBAYASHI_EGFR_SIGNALING_6HR_DN(LS, -7), FARMER_BREAST_CANCER_CLUSTER_5 (LS, -15); cultured astroglia vs. *in vivo* astrocytes (CS, -11); Sec. lymphoid organs (TS, -7)
R	DNA replication	117/5	*non-mBL** *intermediate B*		positive regulation of DNA replication (BP, -7), stored secretory granule (CC, -4); 3',5'-cyclic-AMP phosphodiesterase activity (MF, -5); FAS_PATHWAY (BC, -6); EFFECTS_OF_PIP2_HYDROLYSIS (RE, -6); regulation of protein binding (BP, -8);	Bone marrow (TS, -5); CHIANG_LIVER_CANCER_SUBCLASS_UNANNOTATED_UP (LS, -6); CROONQUIST_NRAS_SIGNALING_UP (LS, -6); MATTIOLI_MULTIPLE_MYELOMA_WITH_14Q32_TRANSLOCATIONS (LS, -5); LIU_NASOPHARYNGEAL_CARCINOMA (LS, -5)

Table S1. *Cont.*

Spot [a]	Short name	#G/#MG [b]	Up [c]	Down [c]	GO and pathway sets [d]	Tissue and disease sets [e]
W		43/5	*non-mBL**	*mBL*, intermediate A & B*	endoplasmic reticulum (CC, -12); cell redox homeostasis (BP, -6); AHSP_PATHWAY (BC, -6);	SCHLOSSER_MYC_TARGETS_AND_SERUM_ RESPONSE_DN (LS, -5); NIKOLSKY_BREAST_ CANCER_16P13_AMPLICON (LS, -5); IIZUKA_LIVER_CANCER_PROGRESSION_G1_G2_DN (LS, -5)
G	fibrino-lysis	131/1			extracellular space (CC, -15); negative regulation of endopeptidase activity (BP, -14); FIBRINOLYSIS_PATHWAY (BC, -12); microsome (CC, -11)	
B		120/3			external side of plasma membrane (CC, -5); positive regulation of hormone secretion (BP, -4); heterophilic cell-cell adhesion (BP, -4); melanocyte differentiation (BP, -4)	SENGUPTA_NASOPHARYNGEAL_CARCINOMA_WITH_LMP1 _UP (LS, -4); GNATENKO_PLATELET_SIGNATURE (LS, -4);
C		97/3			OPSINS (RE, -5); glutamate receptor activity (MF, -5); STEM_PATHWAY (BC, -4)	GAL_LEUKEMIC_STEM_CELL_UP (LS, -4); YAGI_AML_RELAPSE_PROGNOSIS (LS, -4)
S	tonsil	53/3			keratinocyte differentiation (BP, -10); desmosom (CC, -10); structural constituent of cytoskeleton (MF, -8)	Tonsil (TS, -9); Epithelium (TS, -9); ROY_WOUND_BLOOD_VESSEL_DN (LS, -9);
T	tonsil	83/4			homophilic cell adhesion (BP, -5); extracellular region (CC, -5)	Tonsil (TS, -3);
V		55/6	*intermediate A & B*			MATTIOLI_MULTIPLE_MYELOMA_SUBGROUPS (LS, -9), MATTIOLI_MULTIPLE_MYELOMA_WITH_14Q32_TRANSLOC ATIONS (LS, -4)

[a] Sets are assigned using the letter-nomenclature introduced in the main paper; [b] Number of genes/number of metagenes in the spot; [c] Cancer subtypes showing up- or downregulation of the respective spot; [d] Top enriched gene sets from the categories biological process (BP), molecular function (MF), cellular component (CC), reactome (RE), biocarta (BC), KEGG (KG). Enrichment is estimated using the *p*-value of the right-tailed Fishers exact test. The table lists the name of the gene set and the set category and the log10 p-value in the brackets; [e] Top enriched genesets from the categories ‘literature sets’ (LS), cell systems (CS), tissue sets (TS).

Figure S6. GSZ-profiles and population maps are shown for selected gene sets accumulating in the subtype specific overexpression spots as indicated by the red ellipses.

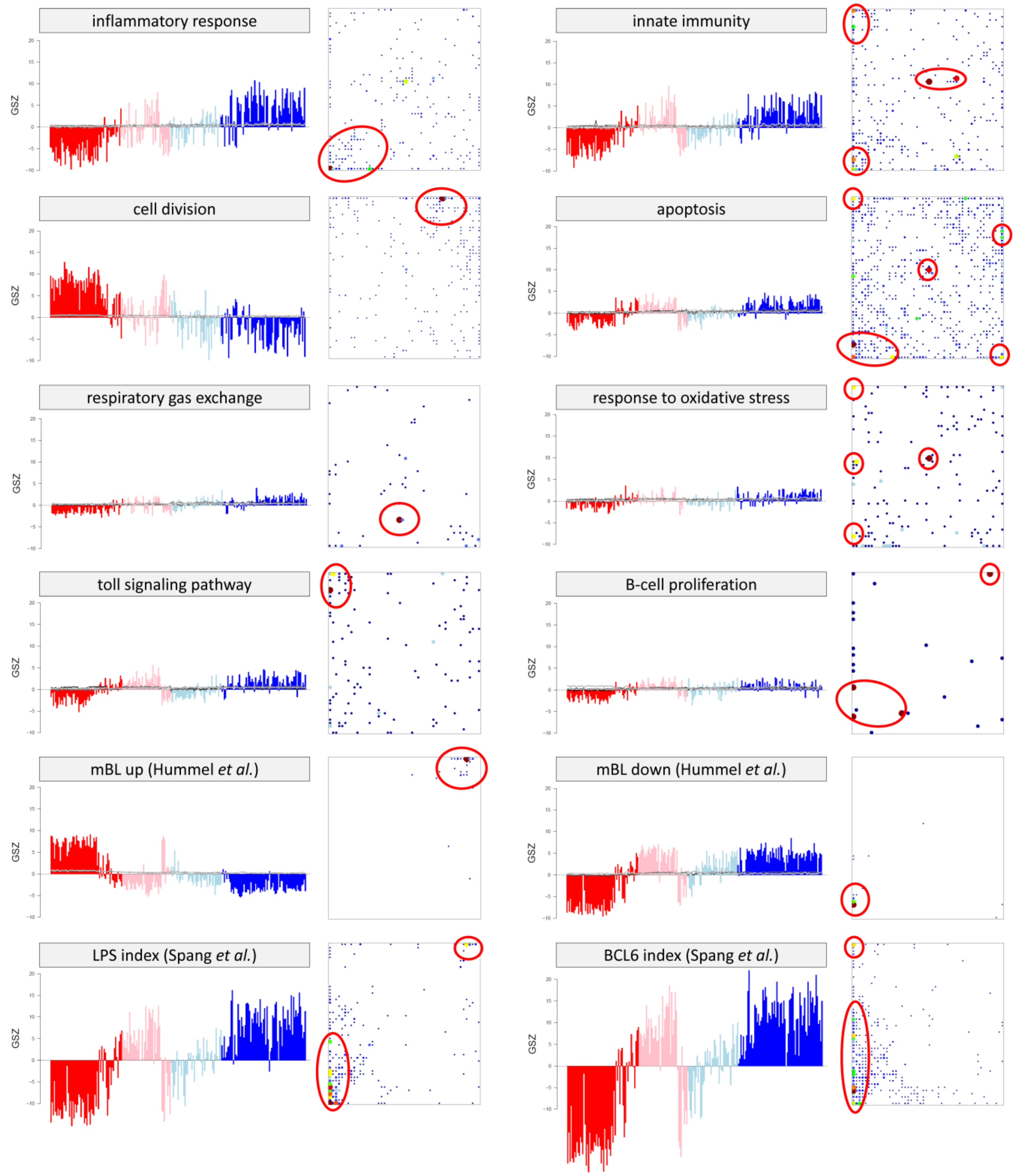

Figure S6. *Cont.*

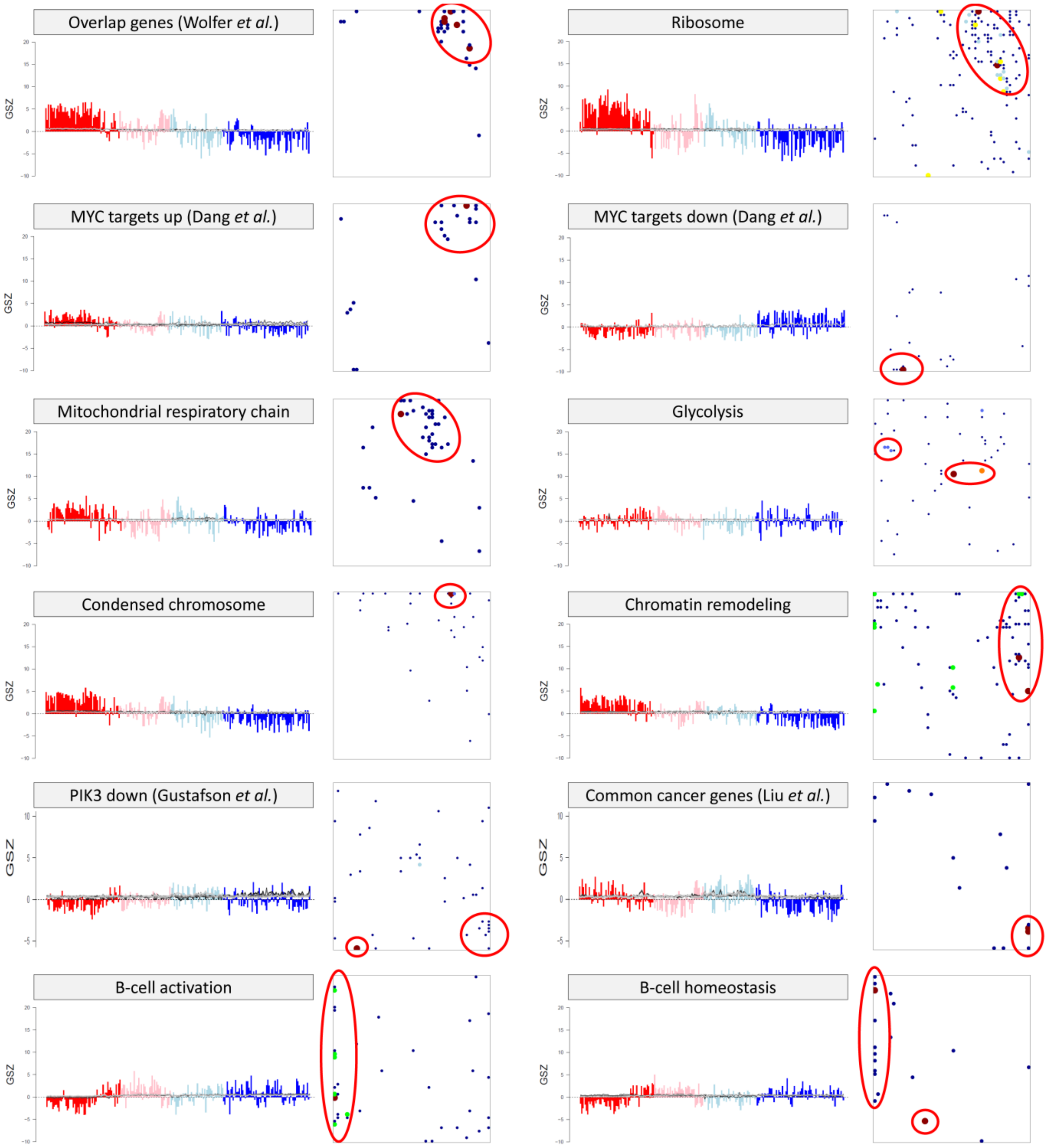

Note that the expression levels of these hallmark-sets are on an intermediate level in most of the samples of the *intermediate* subtypes (e.g., see the sets 'inflammatory response' and 'cell division' in Figure S6). On the other hand, the signature sets of the mBL and non-mBL subtypes (see 'mBL_up' and 'mBL_down' in Figure S6) suggest a *non-mBL**-like behavior of the *intermediate* subtypes which both change in concert with the *non-mBL** samples. In contrast, other gene sets obtained from pathway activation experiments in the B-cell lymphoma context (see 'LPS index' and 'BCL6 index' in Figure S6) reveal a differential behavior of the *intermediate* subtypes. The latter gene sets show similar

profiles as the GO-gene sets 'innate immunity', 'toll signaling pathway' and 'apoptosis' suggesting the selective activation of pathways related to innate immunity responses.

In addition to the fact that each of the *intermediate* subtypes is characterized by two expression modules, these subtypes also show a more complex co-expression behavior compared with that of the *mBL*/non-mBL** subtypes: Modules up-regulated in the *intermediate* subtypes positively as well as negatively correlate with the *mBL** and the *non-mBL** modules, respectively (see Figure S5). Hereby, the two modules of the *intermediate A* subtype behave differently: The 'toll-like receptor' module 'J' switches antagonistically with the two modules up-regulated in the *intermediate B* subtype, whereas the second module 'U' switches in concert with the *intermediate B*, the *non-mBL**, and, of course, the *intermediate A* modules, respectively. Thus, the activation of processes related to 'humoral immunity' seems to be a common property of these three subtypes. In general, these network characteristics show an underlying complex behavior reflecting the heterogeneity of the *intermediate* subtypes on the level of gene expression.

Enrichment analysis also shows that gene sets obtained in the context of different cancer studies enrich in the *mBL** and *non-mBL** modules which supports our result that these two subtypes in final consequence reflect hallmarks of cancer (see the last column in Table S1). In particular, the *mBL** modules enrich cancer gene sets associated to poor survival prognosis due to deregulation of the MYC oncogene [6] (see Figure S6). Expression changes of selected MYC-targets agree with this result [7]: Activated MYC targets accumulate in the same region of the map as the poor prognosis genes together with GO sets related to transcriptional and translational activity. This result supports the view that MYC acts as a general amplifier of gene activity governed by chromatin remodeling [8]. Interestingly, the expression of gene sets related to chromatin structure (see 'condensed chromosome' and 'chromatin remodeling' in Figure S6) change in concert with the MYC-target, poor prognosis and mBL signature sets.

Also, the spot modules up-regulated in samples of the *intermediate* subtypes enrich gene sets from other cancer studies. Interestingly, common cancer genes [9] are found in spot 'Q' related to the *intermediate B* subtype. This set contains genes of the PIK3 signaling-pathway. An independently obtained set of genes deactivated by PIK3 are specifically up-regulated in samples of the *intermediate B* subtype ('PIK3 down', Figure S6).

These puzzling results require further studies. It becomes however clear that gene regulation in each of the subtypes and especially in the two *intermediate* classes splits into more than one mode which can co-regulate or anti-co-regulate each with another. Hence, the decomposition into four subtypes further diversifies into different modes per subtype on the level of gene regulation which, in turn, reflects driving effects on the genetic and epigenetic levels.

5.2. Comparison with Healthy Cell-of-Origin Controls

The 'tonsil' module (spots 'S' and 'T') is positively and negatively correlated with the *non-mBL** and *mBL** modules, respectively, in our modular network (Figure S5). In other words, the *non-mBL** subtype is more tonsil-like than the *mBL** subtype. To prove this result we included microarray expression data of healthy control samples into our analysis with the final aim to assess the cell of origin characteristics of the new four subtypes defined. Expression data of 23 control samples

(10 tonsils, 13 germinal centers (GC) B-cells) were preprocessed as described in the main paper and combined with the 221 lymphoma samples to train a new SOM. The metadata obtained was then utilized to generate the correlation network (CN) shown in Figure S7. It clearly reveals close similarity of tonsils to *non-mBL** and partly to *intermediate B* lymphomas. Also, GC B-cells are very similar to *intermediate B* showing however a closer similarity to *mBL** lymphoma. These findings support the enrichment of lymphoma of the germinal center B-cell (GCB) type in the *mBL**, *non-mBL** and, to a lesser extent, *intermediate B* subtypes (see Table 1 in the main paper).

Figure S7. Correlation network (CN) of the combined lymphoma and healthy control SOM.

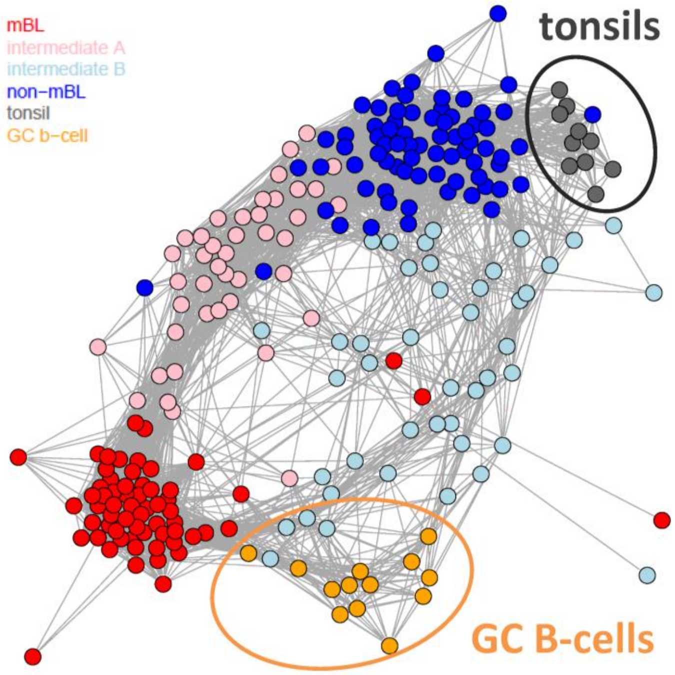

On the other hand, samples from the *intermediate A* subtype are dissimilar to the gene expression signatures of tonsils and GC B-cells supporting their preferential assignment to lymphoma of the activated B-cell (ABC) type as discussed in the main paper. In further support of this result we found that gene set signatures of 'activated B-cells' and of 'B-cell homeostasis' clearly enrich in the region of spots 'U' and 'J' up-regulated in the *intermediate A* subtype (Figure S6). Their expression profiles clearly up-regulate in samples of this subtype.

References

1. Wirth, H.; Loffler, M.; von Bergen, M.; Binder, H. Expression cartography of human tissues using self organizing maps. *BMC Bioinform.* **2011**, *12*, doi:10.1186/1471-2105-12-306.
2. Wirth, H. Analysis of Large-Scale Molecular Biological Data Using Self-Organizing Maps. Available online: http://www.qucosa.de /fileadmin/data/qucosa/documents/10129/Dissertation%20Henry%20Wirth.pdf (accessed on 14 November 2013).
3. Kohonen, T. *Self Organizing Maps*; Springer: Berlin, Heidelberg, Germany, New York, NY, USA, 1995.

4. Hopp, L.; Wirth, H.; Fasold, M.; Binder, H. Portraying the expression landscapes of cancer subtypes: A glioblastoma multiforme and prostate cancer case study. *Syst. Biomed.* **2013**, *1*, in press.
5. Zhang, B.; Horvath, S. A general framework for weighted gene co-expression network analysis. *Stat. Appl. Genet. Mol. Biol.* **2005**, *4*, doi:10.2202/1544-6115.1128.
6. Wolfer, A.; Wittner, B.S.; Irimia, D.; Flavin, R.J.; Lupien, M.; Gunawardane, R.N.; Meyer, C.A.; Lightcap, E.S.; Tamayo, P.; Mesirov, J.P.; *et al.* MYC regulation of a "poor-prognosis" metastatic cancer cell state. *Proc. Natl. Acad. Sci. USA* **2010**, *107*, 3698–3703.
7. Dang, C.V. Enigmatic MYC conducts an unfolding systems biology symphony. *Genes Cancer* **2010**, *1*, 526–531.
8. Nie, Z.; Hu, G.; Wei, G.; Cui, K.; Yamane, A.; Resch, W.; Wang, R.; Green, D.R.; Tessarollo, L.; Casellas, R.; *et al*. c-Myc is a universal amplifier of expressed genes in lymphocytes and embryonic stem cells. *Cell* **2012**, *151*, 68–79.
9. Lu, Y.; Yi, Y.; Liu, P.; Wen, W.; James, M.; Wang, D.; You, M. Common human cancer genes discovered by integrated gene-expression analysis. *PLoS One* **2007**, *2*, e1149.

© 2013 by the authors; licensee MDPI, Basel, Switzerland. This article is an open access article distributed under the terms and conditions of the Creative Commons Attribution license (http://creativecommons.org/licenses/by/3.0/).
