# Supplementary material for: Portraying the Expression Landscapes of B-Cell Lymphoma-Intuitive Detection of Outlier Samples and of Molecular Subtypes
Source: Biology (Basel). 2013 Dec 2;2(4):1411–37. doi: 10.3390/biology2041411 (PMC4009791; doi:10.3390/biology2041411)
Supplement: Supplementary File 1 — Supplementary (ZIP, 8169 KB) [file biology-02-01411-s001.zip › supplementary/additional file 02.pdf]

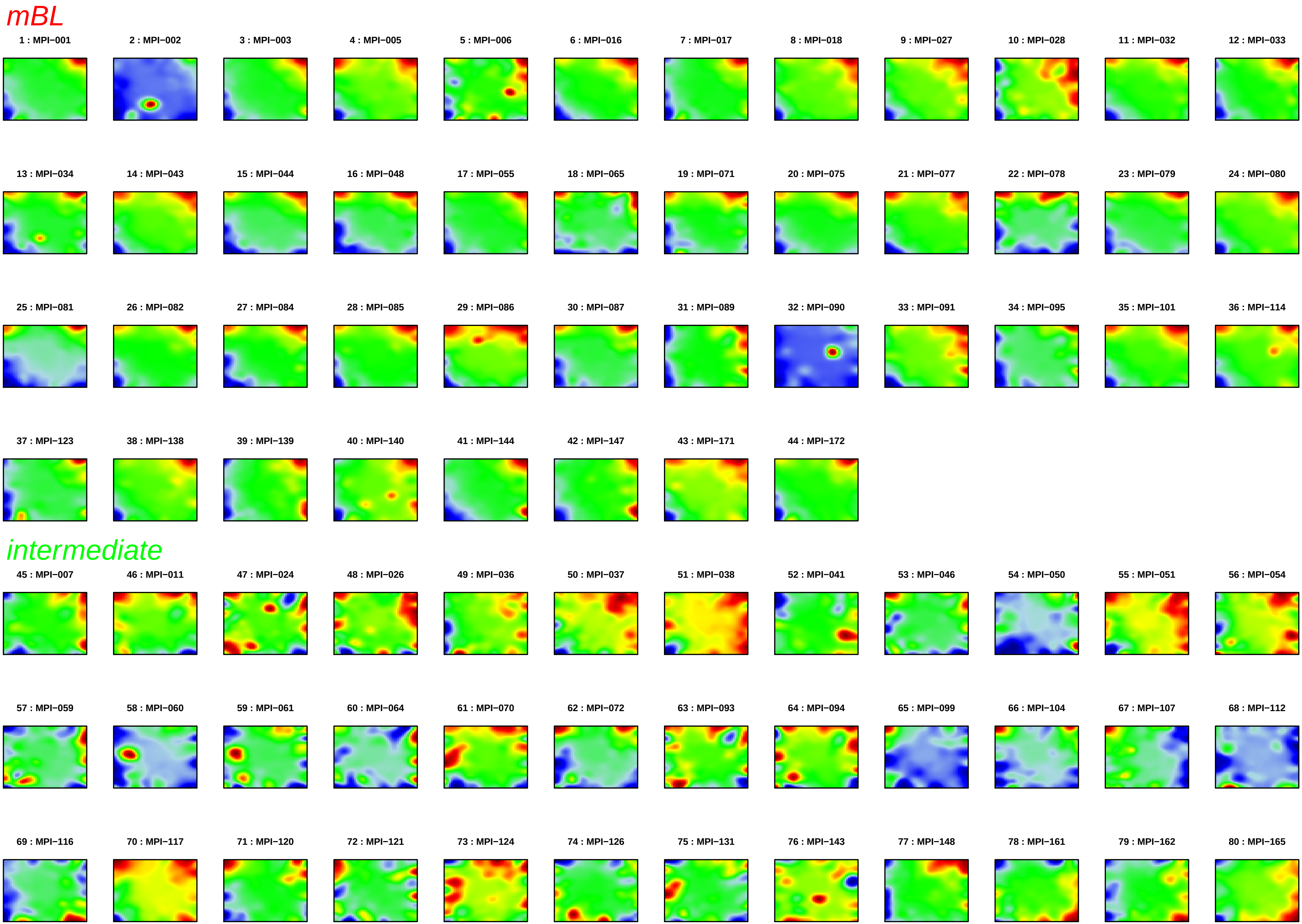
mBL
1 : MPI−001
2 : MPI−002
3 : MPI−003
4 : MPI−005
5 : MPI−006
6 : MPI−016
7 : MPI−017
8 : MPI−018
9 : MPI−027
10 : MPI−028
11 : MPI−032
12 : MPI−033
13 : MPI−034
14 : MPI−043
15 : MPI−044
16 : MPI−048
17 : MPI−055
18 : MPI−065
19 : MPI−071
20 : MPI−075
21 : MPI−077
22 : MPI−078
23 : MPI−079
24 : MPI−080
25 : MPI−081
26 : MPI−082
27 : MPI−084
28 : MPI−085
29 : MPI−086
30 : MPI−087
31 : MPI−089
32 : MPI−090
33 : MPI−091
34 : MPI−095
35 : MPI−101
36 : MPI−114
37 : MPI−123
38 : MPI−138
39 : MPI−139
40 : MPI−140
41 : MPI−144
42 : MPI−147
43 : MPI−171
44 : MPI−172
intermediate
45 : MPI−007
46 : MPI−011
47 : MPI−024
48 : MPI−026
49 : MPI−036
50 : MPI−037
51 : MPI−038
52 : MPI−041
53 : MPI−046
54 : MPI−050
55 : MPI−051
56 : MPI−054
57 : MPI−059
58 : MPI−060
59 : MPI−061
60 : MPI−064
61 : MPI−070
62 : MPI−072
63 : MPI−093
64 : MPI−094
65 : MPI−099
66 : MPI−104
67 : MPI−107
68 : MPI−112
69 : MPI−116
70 : MPI−117
71 : MPI−120
72 : MPI−121
73 : MPI−124
74 : MPI−126
75 : MPI−131
76 : MPI−143
77 : MPI−148
78 : MPI−161
79 : MPI−162
80 : MPI−165

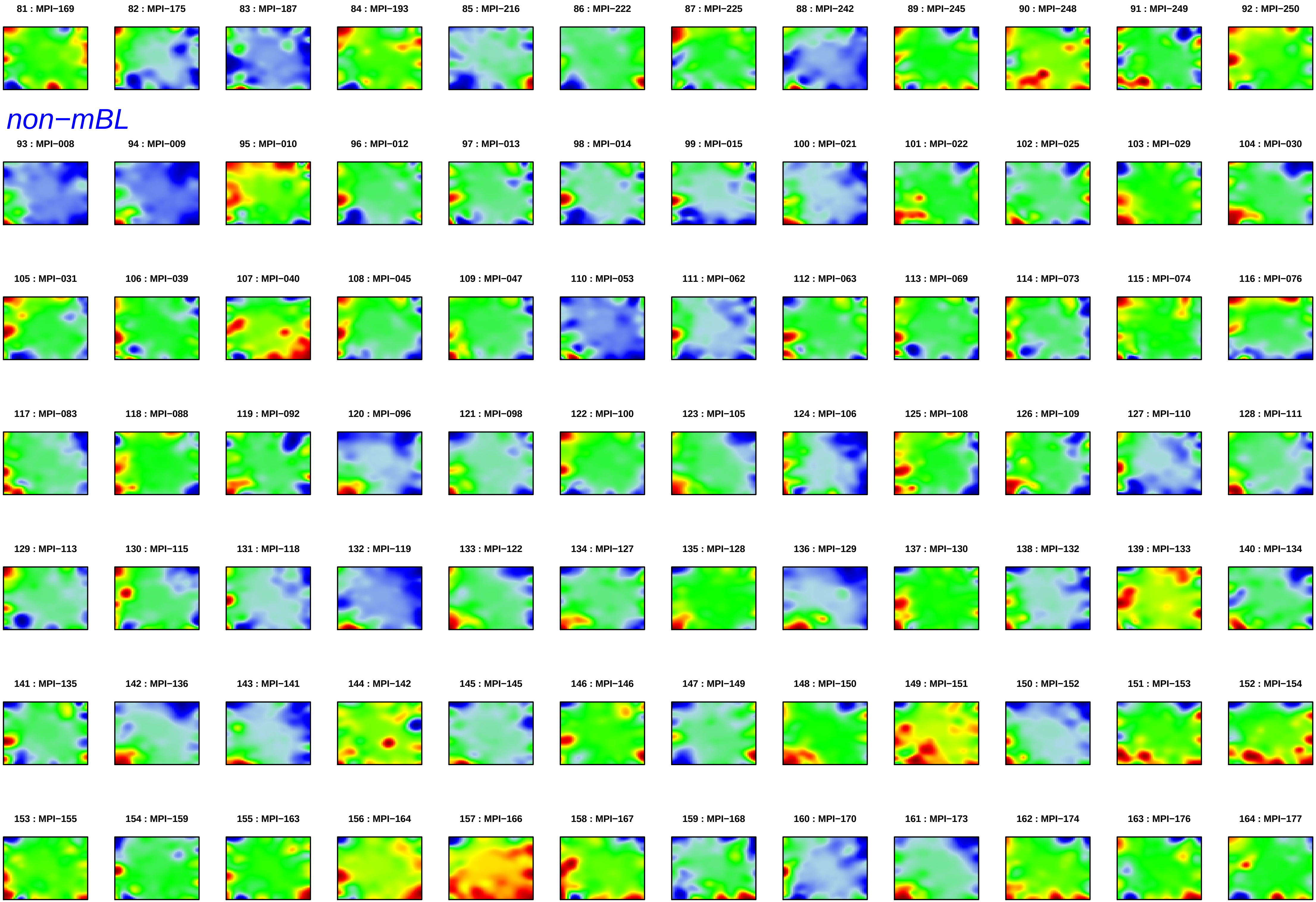
81 : MPI−169
82 : MPI−175
83 : MPI−187
84 : MPI−193
85 : MPI−216
86 : MPI−222
87 : MPI−225
88 : MPI−242
89 : MPI−245
90 : MPI−248
91 : MPI−249
92 : MPI−250
non−mBL
93 : MPI−008
94 : MPI−009
95 : MPI−010
96 : MPI−012
97 : MPI−013
98 : MPI−014
99 : MPI−015
100 : MPI−021
101 : MPI−022
102 : MPI−025
103 : MPI−029
104 : MPI−030
105 : MPI−031
106 : MPI−039
107 : MPI−040
108 : MPI−045
109 : MPI−047
110 : MPI−053
111 : MPI−062
112 : MPI−063
113 : MPI−069
114 : MPI−073
115 : MPI−074
116 : MPI−076
117 : MPI−083
118 : MPI−088
119 : MPI−092
120 : MPI−096
121 : MPI−098
122 : MPI−100
123 : MPI−105
124 : MPI−106
125 : MPI−108
126 : MPI−109
127 : MPI−110
128 : MPI−111
129 : MPI−113
130 : MPI−115
131 : MPI−118
132 : MPI−119
133 : MPI−122
134 : MPI−127
135 : MPI−128
136 : MPI−129
137 : MPI−130
138 : MPI−132
139 : MPI−133
140 : MPI−134
141 : MPI−135
142 : MPI−136
143 : MPI−141
144 : MPI−142
145 : MPI−145
146 : MPI−146
147 : MPI−149
148 : MPI−150
149 : MPI−151
150 : MPI−152
151 : MPI−153
152 : MPI−154
153 : MPI−155
154 : MPI−159
155 : MPI−163
156 : MPI−164
157 : MPI−166
158 : MPI−167
159 : MPI−168
160 : MPI−170
161 : MPI−173
162 : MPI−174
163 : MPI−176
164 : MPI−177

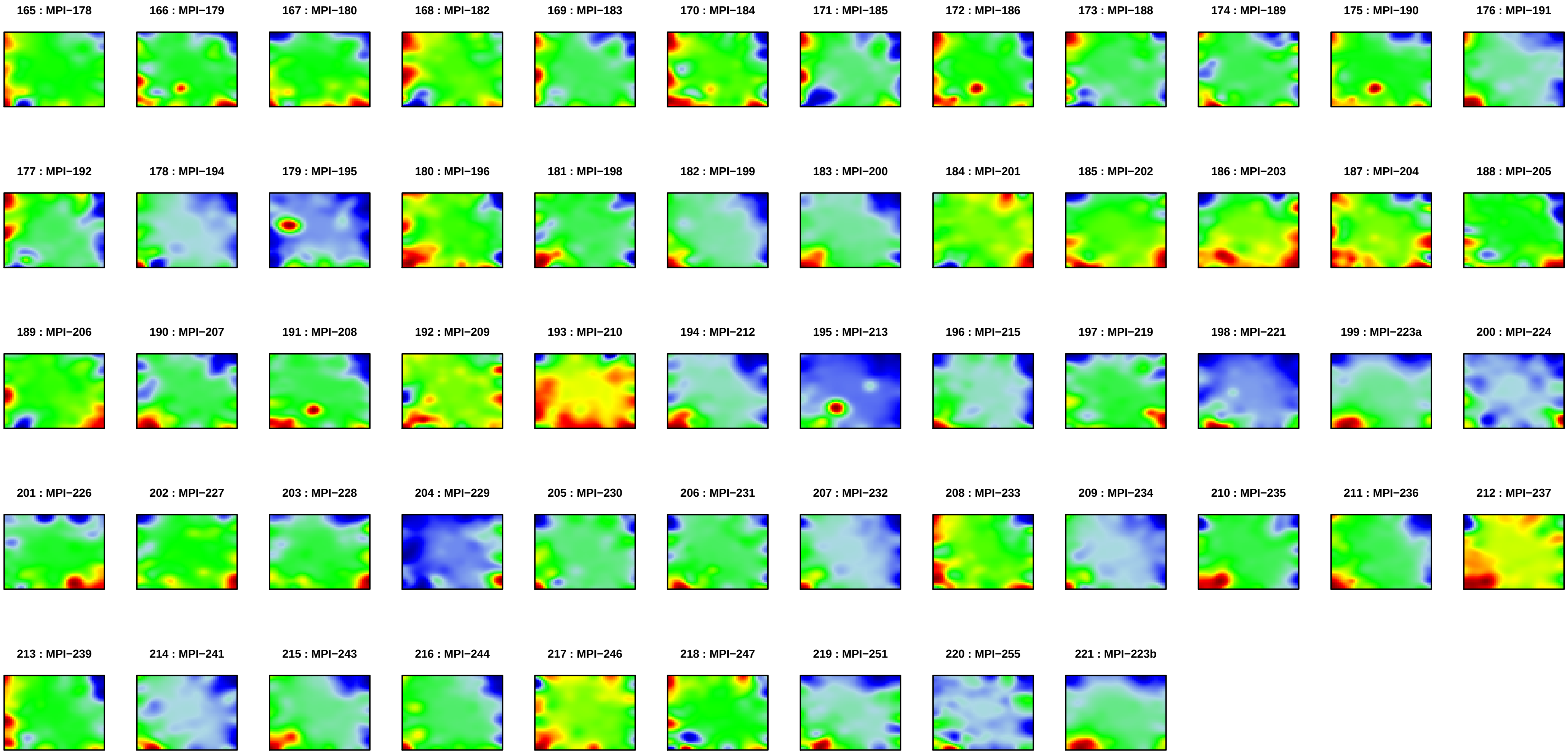
165 : MPI−178
166 : MPI−179
167 : MPI−180
168 : MPI−182
169 : MPI−183
170 : MPI−184
171 : MPI−185
172 : MPI−186
173 : MPI−188
174 : MPI−189
175 : MPI−190
176 : MPI−191
177 : MPI−192
178 : MPI−194
179 : MPI−195
180 : MPI−196
181 : MPI−198
182 : MPI−199
183 : MPI−200
184 : MPI−201
185 : MPI−202
186 : MPI−203
187 : MPI−204
188 : MPI−205
189 : MPI−206
190 : MPI−207
191 : MPI−208
192 : MPI−209
193 : MPI−210
194 : MPI−212
195 : MPI−213
196 : MPI−215
197 : MPI−219
198 : MPI−221
199 : MPI−223a
200 : MPI−224
201 : MPI−226
202 : MPI−227
203 : MPI−228
204 : MPI−229
205 : MPI−230
206 : MPI−231
207 : MPI−232
208 : MPI−233
209 : MPI−234
210 : MPI−235
211 : MPI−236
212 : MPI−237
213 : MPI−239
214 : MPI−241
215 : MPI−243
216 : MPI−244
217 : MPI−246
218 : MPI−247
219 : MPI−251
220 : MPI−255
221 : MPI−223b
