# Supplementary figures and images for: Portraying the Expression Landscapes of B-Cell Lymphoma-Intuitive Detection of Outlier Samples and of Molecular Subtypes
Source: Biology (Basel). 2013 Dec 2;2(4):1411–37. doi: 10.3390/biology2041411 (PMC4009791; doi:10.3390/biology2041411)

*mBL* \*

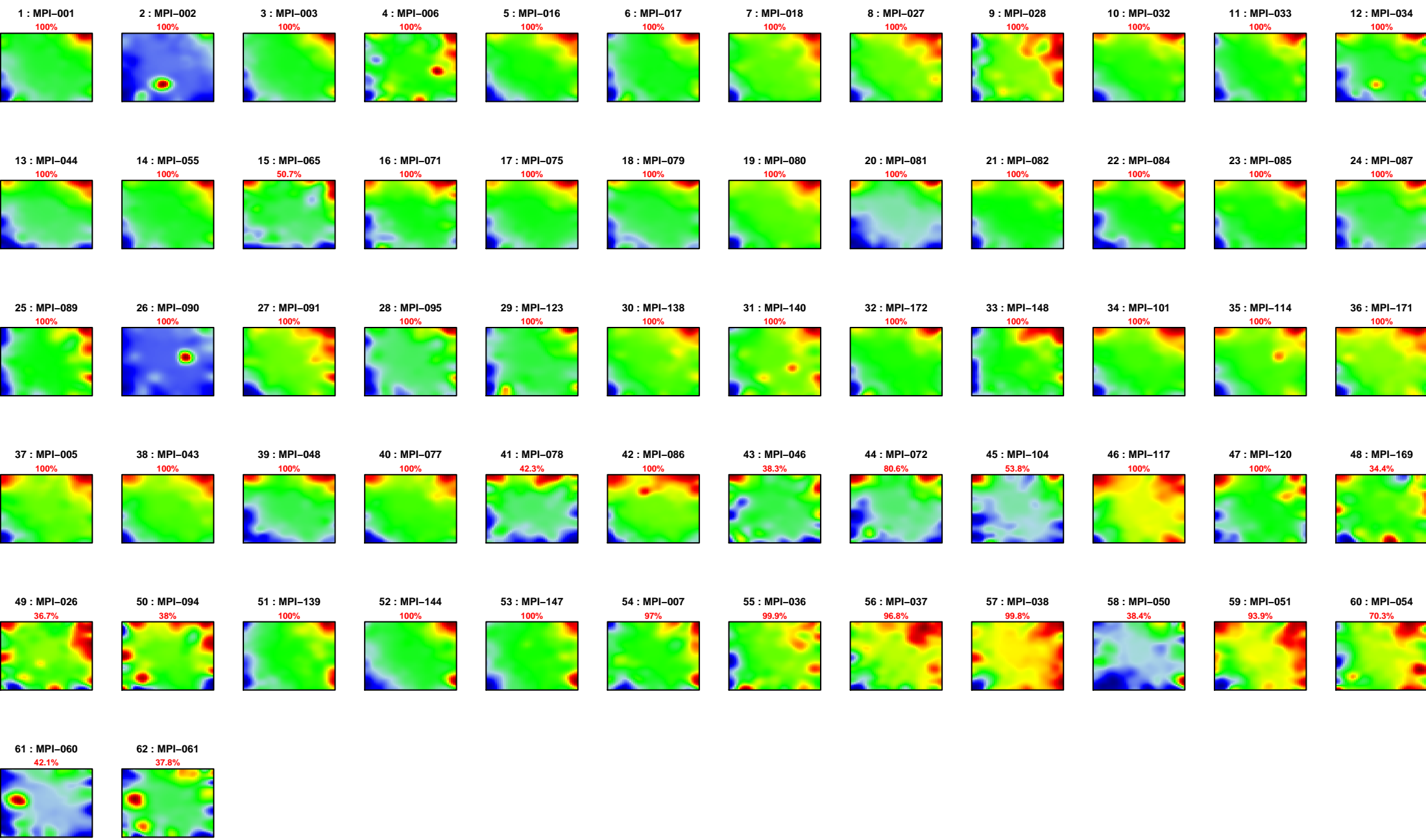

*intermediate A*

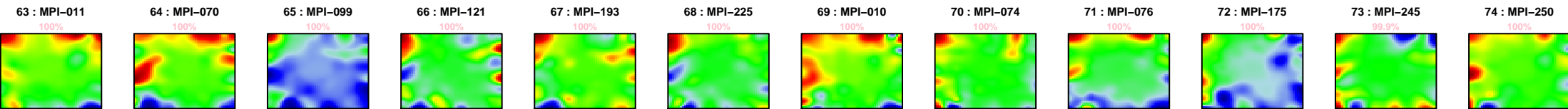

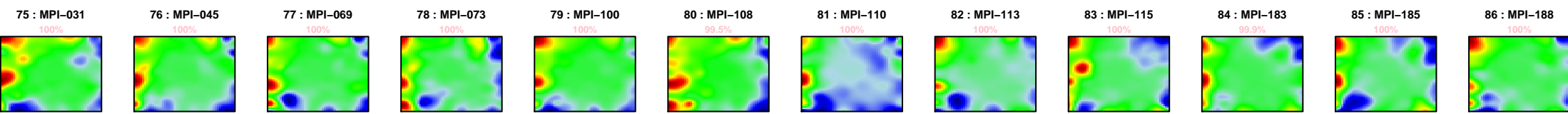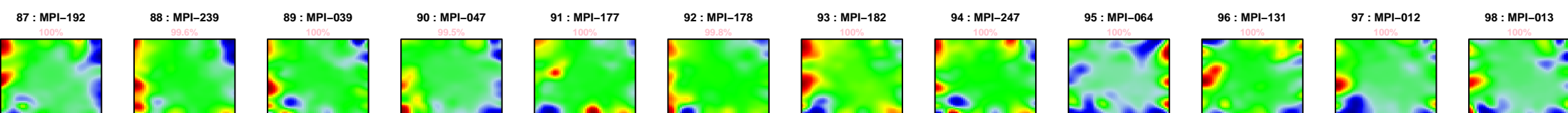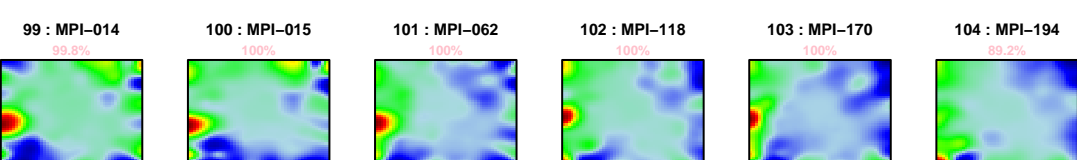

*intermediate B*

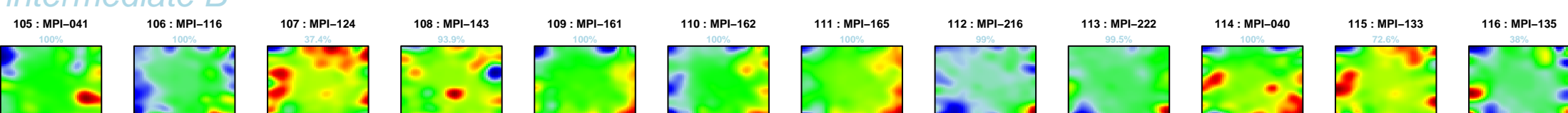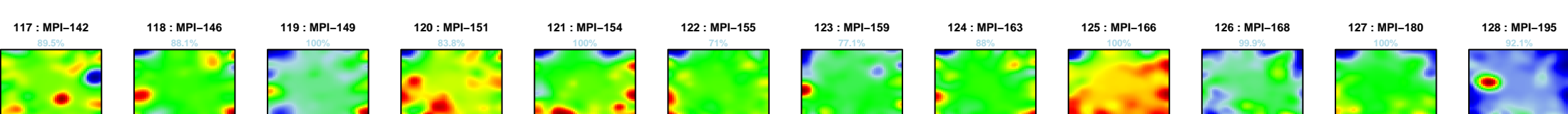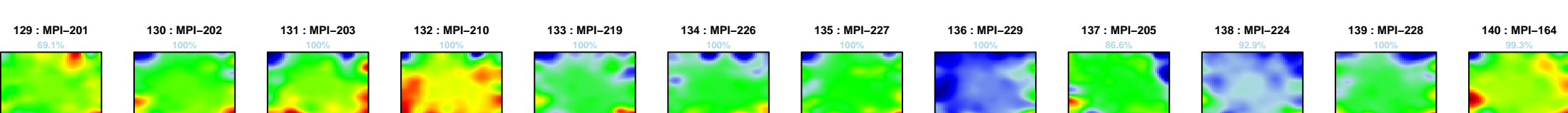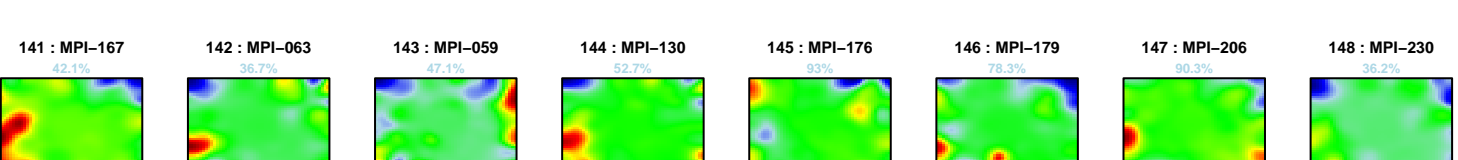

*non-mBL\**

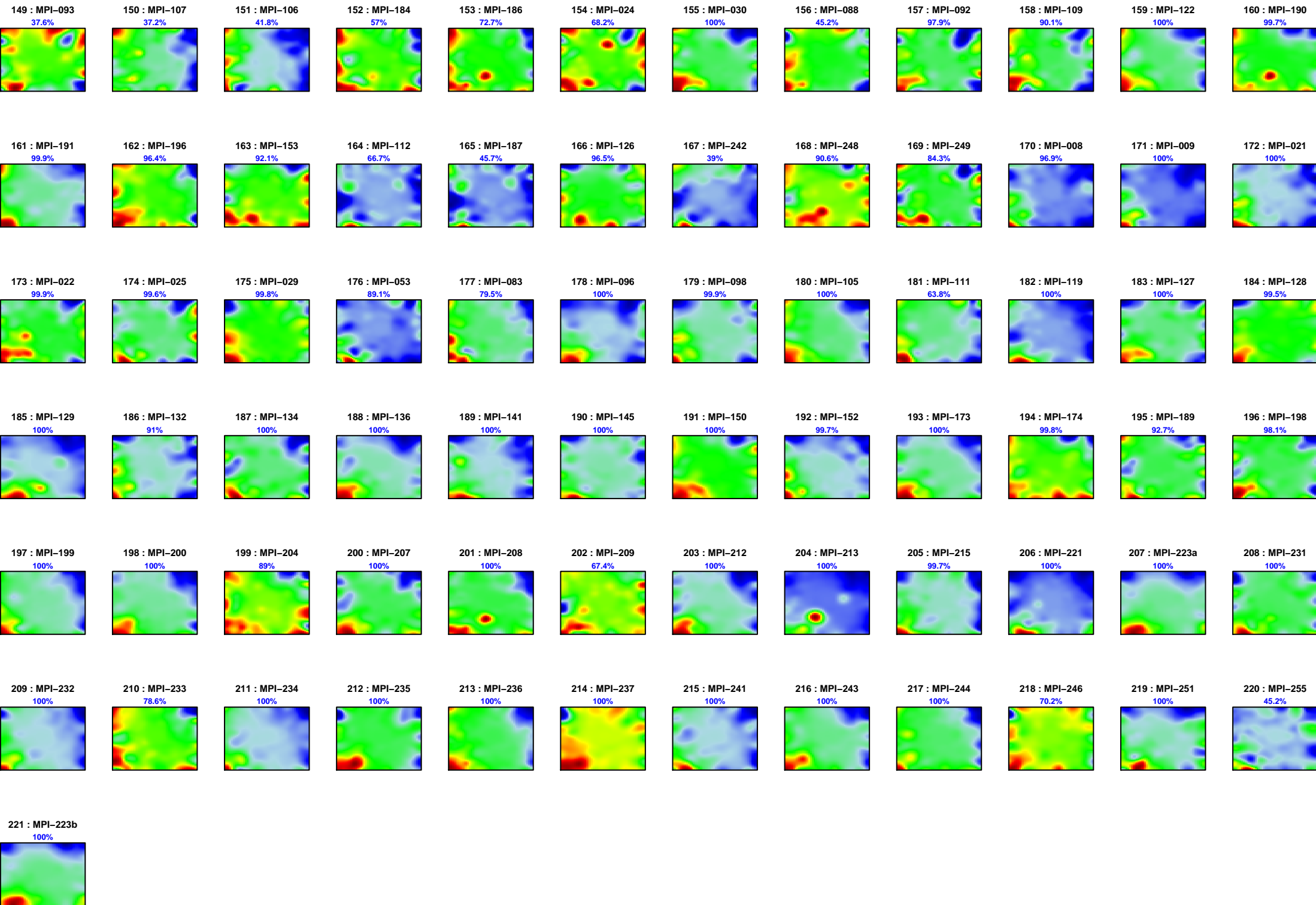

Supplement: Supplementary File 1 — Supplementary (ZIP, 8169 KB) [file biology-02-01411-s001.zip › supplementary/additional file 03.pdf]
